# Modulation of the antitumor immune response by cancer-associated fibroblasts: mechanisms and targeting strategies to hamper their immunosuppressive functions

**DOI:** 10.37349/etat.2022.00103

**Published:** 2022-10-27

**Authors:** Jerome Thiery

**Affiliations:** 1 INSERM, UMR 1186, 94800 Villejuif, France; 2 Gustave Roussy Cancer Campus, 94805 Villejuif, France; 3 University Paris Saclay, Faculty of Medicine, 94270 Le Kremlin Bicêtre, France; Istituto Nazionale Tumori “Fondazione Pascale” Via Mariano Semmola, Italy

**Keywords:** Tumor microenvironment, cancer-associated fibroblasts, immune suppression, cancer immunotherapy

## Abstract

Cancer-associated fibroblasts (CAFs) are highly heterogeneous players that shape the tumor microenvironment and influence tumor progression, metastasis formation, and response to conventional therapies. During the past years, some CAFs subsets have also been involved in the modulation of immune cell functions, affecting the efficacy of both innate and adaptive anti-tumor immune responses. Consequently, the implication of these stromal cells in the response to immunotherapeutic strategies raised major concerns. In this review, current knowledge of CAFs origins and heterogeneity in the tumor stroma, as well as their effects on several immune cell populations that explain their immunosuppressive capabilities are summarized. The current development of therapeutic strategies for targeting this population and their implication in the field of cancer immunotherapy is also highlighted.

## Introduction

During the past decades, accumulating evidence has revealed that tumor progression and response to therapies do not only rely on cancer cell genetic or epigenetic alterations but are also controlled by several components of the tumor microenvironment (TME) [1–3]. Indeed, the TME is a complex ecosystem composed of several cell types from endothelial/mesenchymal lineages and of various immune cells embedded in an intricated extracellular matrix (ECM), which enter into a dynamic relationship with tumor cells [2, 4–6]. Of note, over the last years, the TME has emerged also as a crucial regulator that shapes the cellular fate and functions of tumor-infiltrating lymphocytes (TILs), promotes tumor cell evasion from immune cell-mediated cytotoxicity and consequently alters the efficacy of the anti-tumor immune response or potentially immunotherapeutic approaches [7–9]. This last point relies, at least in part, on the ability of tumor cells and the TME components to orchestrate an immunosuppressive landscape, which leads, for example, to the recruitment and differentiation of immunosuppressive cells and ultimately to the inhibition of immune effector/killer cell functions. In particular, within the tumor stroma, fibroblasts that share similarities with fibroblasts activated during tissue injury or by acute or chronic inflammation, also named cancer-associated fibroblasts (CAFs), play a critical role in tumor cell-stroma complex interactions [10–13] and the regulation of the anti-tumor immune response [14–20]. In this review, the cellular, molecular, and biomechanical aspects involved in the immuno-suppressive capabilities of CAFs within the TME are summarized and the latest updates regarding therapeutic targeting of this cell population are highlighted, with potential implications in the field of combined cancer immunotherapies.

## Diversity of CAF origin and heterogeneity in the TME

**Figure 1.**
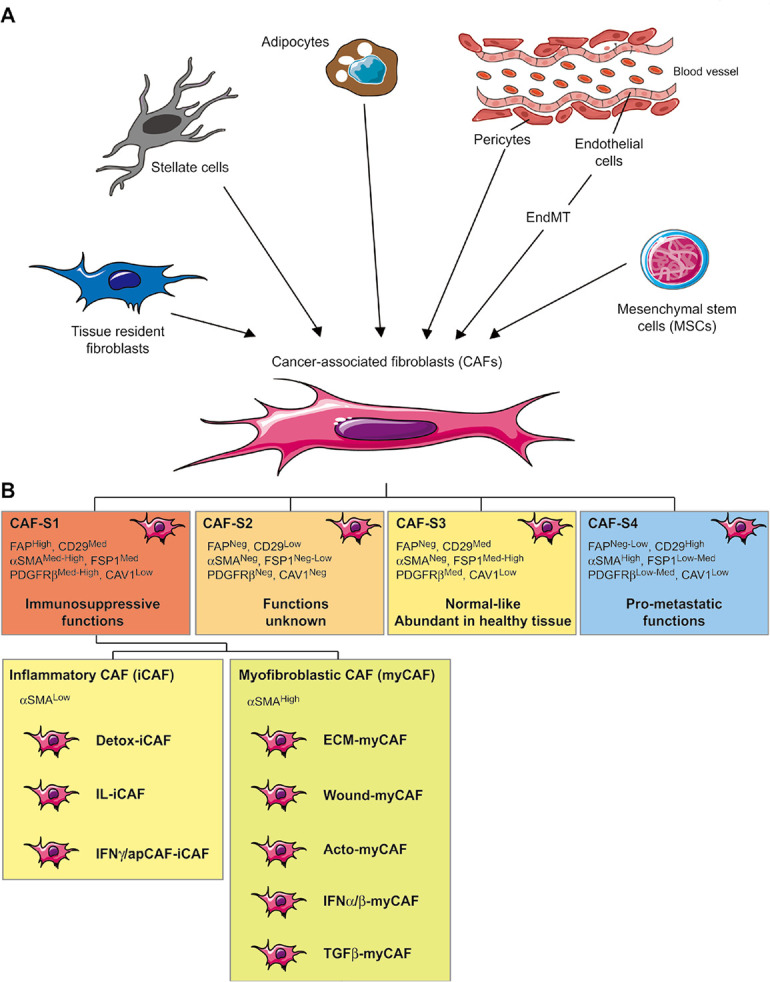
Origins and heterogeneity of CAFs in the TME. A. Schematic representation of CAF origins. CAFs can originate from diverse cell populations through different mechanisms. Local sources of CAFs include activated tissue resident fibroblasts, trans-differentiated endothelial cells resulting from EndMT, and de-differentiated pericytes, adipocytes, or stellate cells. Beyond those local sources, more distant ones can be involved in CAFs recruitment/differentiation in the TME, especially MSCs; B. schematic representation of CAF subsets. Distinct subpopulations of CAFs have been described with the TME. The combined analysis of six CAF markers (FAP, CD29, αSMA, FSP1, PDGFRβ, and CAV1) in breast and ovarian cancer leads to the identification of CAF-S1 to CAF-S4 subtypes. CAF-S1 displays an immune-suppressive function, CAF-S4 promotes invasion and metastasis formation and CAF-S2/-S3 resembles normal fibroblasts. More recently, single-cell RNA sequencing allowed the description of two different subsets of the CAF-S1 population, referred to as myCAF and iCAF. Within these two populations, IL-iCAF (IL-signaling), IFNγ/ap-iCAF (IFNγ-related/antigen presenting pathway), detox-iCAF (detoxification pathway), ECM-myCAF (ECM proteins), TGFβ-myCAF (TGFβ-dependent pathway), wound-myCAF (wound-healing signaling), IFNα/β-myCAF (IFNα/β-related pathway) and acto-myCAF (acto-myosin signaling) have been identified *Note.* Adapted from “Alteration of the antitumor immune response by cancer-associated fibroblasts,” by Ziani L, Chouaib S, Thiery J. Front Immunol. 2018;9:414 (https://doi.org/10.3389/fimmu.2018.00414). © 2018 Ziani, Chouaib and Thiery.

In normal tissue, spindle-shaped, interstitial cells lacking epithelial (cytokeratin^–^, E-cadherin^–^), endothelial (CD31^–^), and immune cell (CD45^–^) markers but from a mesenchymal (vimentin^+^) lineage are usually identified as resting fibroblasts, which display only negligible metabolic and transcriptional activities [11]. On the opposite, following tissue damages and subsequent repair or acute/chronic inflammation [21, 22], fibroblasts can become activated and exhibit contractile activity, exert physical forces to modify tissue architecture, acquire proliferation and migration properties and become transcriptionally active leading to elevated secretion of cytokines, chemokines and ECM components [21, 23, 24]. This process referred to as “wound healing response” is crucial for normal tissue homeostasis but is hijacked by cancer cells to favor their proliferation, survival, or invasive capabilities [11, 25]. Indeed, several studies have demonstrated that tumor cells can activate resident fibroblasts or promote trans-differentiation of other cell populations within the TME that lead to CAF generation [26, 27], which represent one of the most abundant stromal cell populations of several carcinomas including breast, prostate, pancreatic, esophageal and colon cancers [28]. In the context of cancer, several growth factors and cytokines released by either cancer or infiltrating immune cells are key determinants of CAF generation within the TME. For example, transforming growth factor-β (TGFβ), platelet-derived growth factor (PDGF), epidermal growth factor (EGF), fibroblast growth factor (FGF), reactive oxygen species (ROS), interleukin-1β (IL-1β), and IL-6 or lysophosphatidic acid are important determinants of CAF generation within the TME [29–34]. Interestingly, vitamin A or D deficiency can also promote CAF differentiation under certain circumstances [35–37]. Moreover, it is important to note that CAFs can originate from quiescent resident fibroblasts present within the TME, which is probably the main source of this cell population but can also differentiate from other cell populations (Figure 1A). In particular, endothelial-to-mesenchymal transition (EndMT) has been linked to the trans-differentiation of endothelial cells to CAF-like cells [38, 39]. Similarly, perivascular cells, named pericytes, can also de-differentiate into CAFs [40]. Moreover, in breast cancer, adipocytes were shown to de-differentiate into CAFs [41–43], and in pancreas or liver tumors, stellate cells, involved in fibrosis, are probably an important source of CAFs [44, 45]. Finally, mesenchymal stem cells (MSCs), can be attracted from the bone marrow into the TME before their differentiation into CAFs [42, 46–51]. Together with the diversity of “activation” signals, these various origins undoubtedly represent an important determinant that contributes to the heterogeneity of CAFs, which is also highlighted by the diversity of markers used to identify them. This includes fibroblast-activation protein (FAP), α-smooth muscle actin (αSMA), PDGF receptors (PDGFRs), fibroblast-specific protein-1 (FSP1/S100A4), periostin (POSTN), neuron-glial antigen-2 (NG-2), podoplanin (PDPN), desmin, tenascin-C (TN-C), CD90, integrin β-1 (ITGB1/CD29), discoidin domain-containing receptor 2 (DDR2) or caveolin-1 (CAV1) [25, 28, 52–60]. However, none of these proteins is unequivocally specific for activated fibroblasts and consequently cannot be used as a single marker to distinguish CAFs from normal fibroblasts, or even other cell types. Moreover, these markers show distinct expression profiles between CAFs from different tumor types as well within the same tumors, once again reflecting their high degree of heterogeneity within the TME. In this regard, several studies have defined subtypes of CAFs presents in the TME of breast, ovarian, head, neck, and lung cancers or pancreatic ductal carcinoma (PDAC) [57, 61–63]. For example, based on an integrated flow cytometry analysis of FAP, CD29, αSMA, FSP1, PDGFRβ, and CAV1 expression, four different CAFs subsets (named CAF-S1 to -S4) have been identified in different breast and ovarian tumor subtypes and differentially accumulate within the TME [64] (Figure 1B). In highly aggressive human EGF receptor-2 positive (Her2^+^) and triple-negative breast tumors, CAF-S1 (FAP^HIGH^, CD29^MED^, αSMA^MED-HIGH^, FSP1^MED^, PDGFRβ^MED-HIGH^, CAV1^LOW^) and CAF-S4 (FAP^NEG-LOW^, CD29^HIGH^, αSMA^HIGH^, FSP1^LOW-MED^, PDGFRβ^LOW-MED^, CAV1^LOW^) represent the main CAF populations. On the opposite, luminal breast tumors are enriched with CAF-S2 (FAP^NEG^, CD29^LOW^, αSMA^NEG^, FSP1^NEG-LOW^, PDGFRβ^NEG^, CAV1^NEG^). Finally, CAF-S3 (FAP^NEG^, CD29^MED^, αSMA^NEG^, FSP1^MED-HIGH^, PDGFRβ^MED^, CAV1^LOW^) appear like normal fibroblasts also found in healthy tissue. Importantly, these 4 CAF subsets have been validated *in situ* by immunohistochemistry on patient samples [65] and using publicly available single-cell RNASeq (scRNASeq) data, CAF-S1 subtype has been also identified in other tumors including PDAC [66, 67], colorectal [68] or lung cancers [69] and displays inflammation, adhesion and ECM signatures [61] as well as immunosuppressive capabilities. Furthermore, among the CAF-S1 population in PDAC, and more recently in other tumors, two different subsets, αSMA^LOW^ CAF [inflammatory CAF (iCAF)] and αSMA^HIGH^ CAF [myofibroblastic CAF (myCAF)] have been identified [44, 61, 70, 71]. The iCAF subpopulation secretes high levels of proinflammatory/immunomodulatory factors and is distant from the neoplastic cells, while the myCAF subset is located in the proximity of tumor cells and secretes ECM components. Moreover, a recent scRNAseq analysis in breast cancer further identified eight different clusters within the CAF-S1 subpopulation [70]. More specifically, within iCAFs subpopulation, IL-iCAF (IL-signaling), interferon-γ (IFNγ)-iCAF (IFNγ-related pathway), and detox-iCAF (detoxification pathway) have been described. Within myCAFs subpopulation, ECM-myCAF (ECM proteins), TGFβ-myCAF (TGFβ-dependent pathway), wound-myCAF (wound-healing signaling), IFNα/β-myCAF (IFNα/β-related pathway), and acto-myCAF (acto-myosin signaling) have been described. Finally, other subsets of CAF have been defined (see [72–74] for review) including a subpopulation of antigen-presenting CAF (apCAF), expressing a high level of major histocompatibility complex (MHC) class II molecules and CD74 [66], which is probably similar to the IFNγ-iCAF subset previously described.

In summary, many CAF subsets and clusters have been recently described, with a continuously increasing complexity [75]. Nevertheless, two important points to note are the relative proportion of the CAF-S1 cluster within the sequenced cell from the scRNAseq studies mentioned above and the presence of CAF-S1 within multiple tumor types, confirming the relevance of this subset in the field of immunosuppression with potential implication for immunotherapy.

## Impact of CAFs on the antitumor immune response

**Figure 2.**
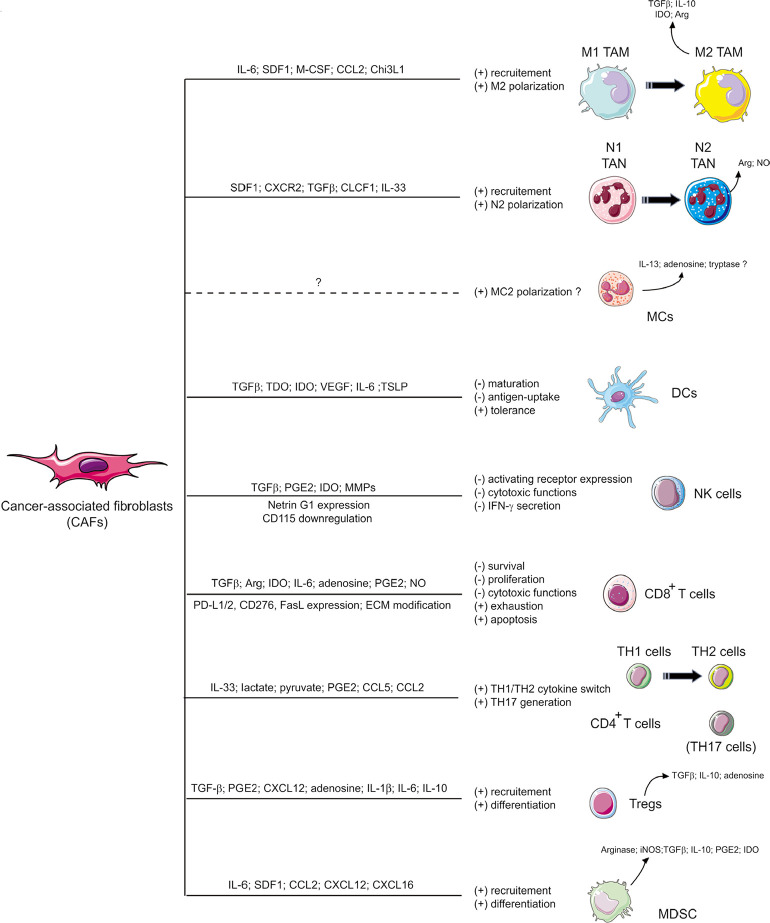
Schematic representation of CAFs-dependent immunosuppression. CAFs shape the tumor immune microenvironment and influence both the innate and adaptive anti-tumor immune response. CAFs are involved in the recruitment of innate immune cells, such as TAMs, and tumor-associated neutrophils (TANs), and promote their acquisition of an immunosuppressive phenotype (M2 and N2 respectively). CAFs also affect the cytotoxic function and cytokine production of natural killer (NK) cells and activate MCs with a potential immunosuppressive phenotype. CAFs also promote the recruitment and differentiation of myeloid-derived suppressor cells (MDSCs) and regulatory T cells (Tregs) and interfere with the maturation and function or dendritic cells (DCs). CAFs have also the ability to influence CD4^+^ Th lymphocytes, favoring tumor-promoting Th2 and Th17 responses, and reduce the activation, functions, and survival of CD8^+^ cytotoxic T cells. MCs: mast cells; CXCR2: C-X-C chemokine receptor 2; CLCF1: cardiotrophin-like cytokine factor 1; TDO: tryptophan 2,3-dioxygenase; TSLP: thymic stromal lymphopoietin; PD-L1: programmed death ligand 1; iNOS: inducible NO synthase; (+): induction; (–): inhibition *Note.* Adapted from “Alteration of the antitumor immune response by cancer-associated fibroblasts,” by Ziani L, Chouaib S, Thiery J. Front Immunol. 2018;9:414 (https://doi.org/10.3389/fimmu.2018.00414). © 2018 Ziani, Chouaib and Thiery.

In the TME, CAFs enter into dynamic crosstalk with tumor cells and/or other TME components and are an important source of several proteins such as ECM components or ECM-remodeling enzymes [e.g., collagens, matrix metallo-proteinases (MMPs)], chemokines [e.g., chemokine C-X-C motif ligand 12 (CXCL12)/stromal cell-derived factor-1 (SDF1)] or chemokine ligands [e.g., C-C motif chemokine ligand 2 (CCL2)/monocyte chemoattractant protein-1 (MCP-1)], angiogenesis-related factors [e.g., vascular endothelial growth factor (VEGF)] and other factors (e.g., TGFβ, EGF, FGF) which are linked to tumor cells proliferation, survival, invasiveness, metabolism reprogramming and stemness [10–13, 25, 28, 76]. Furthermore, and as mentioned above, CAFs have also been involved in the alteration of the anti-tumor immune response by the secretion of several immunomodulators [e.g., TGFβ, IL-1β, IL-6, IL-10, indoleamine-2,3-dioxygenase (IDO), arginase (Arg), CXCL2, CXCL5, CXCL12/SDF1, CCL2/MCP-1, CCL5/regulated upon activation, normal T-cell expressed and secreted (RANTES), VEGF, prostaglandin E2 (PGE2), tumor necrosis factor (TNF) or nitric oxide (NO)], that are key regulators of both innate and adaptive antitumor immune responses [17–19, 77] (Figure 2).

### Alteration of the innate anti-tumor immune response by CAFs

#### Tumor-associated macrophages and CAFs

As a key component of the TME, tumor-associated macrophages (TAMs) play critical roles in the regulation of antitumor immune response. TAMs have been sub-classified into two distinct subtypes. Type I macrophages (or M1) secrete important amounts of pro-inflammatory cytokines and ROS and promote a T-helper 1 (Th1) anti-tumor immune response. On the contrary, type II macrophages (or M2) promote tumor progression and are characterized by the secretion of factors with immune-suppressive activity such as TGFβ, IL-10, Arg, and IDO, which particularly affect cytotoxic CD8^+^ T cell functions [78]. Interestingly, in oral squamous and colorectal cancers, CD163^+^/DC-SIGN^+^ M2 macrophages are the most prominent immune cells in the neighborhood of αSMA^+^, FSP1^+^, and FAP^+^ CAF-rich areas, suggesting a close relationship between these two cell populations, with important consequences on the clinical outcome for patients [79, 80]. Further evidence was provided by several studies which have demonstrated that the recruitment of monocytes into the TME and their differentiation toward M2 subtype macrophages are actively promoted by CAFs [81], especially through their secretion of CXCL12/SDF1, macrophage colony-stimulating factor (M-CSF)/CSF-1, IL-6, CCL2/MCP-1 and chitinase-3-like-1 (Chi3L1)/YKL-40 [82–92]. However, and in an intriguing way, CAFs might also alter TAMs infiltration under certain circumstances, by a FAP-mediated modification of the ECM [93]. Finally, it is important to note that reciprocal crosstalk exists between CAFs and TAMs. Indeed, several studies have suggested that M2 macrophages can regulate CAFs generation, for example by enhancing EMT progression through IL-6 and SDF1 [83], or by influencing the trans-differentiation of MSCs into CAFs [94, 95].

#### TANs and CAFs

Recent evidence indicates that TANs represent a significant component of the TME [96, 97] and several studies have suggested that TANs can be polarized to an N1 anti-tumoral or N2 pro-tumoral subtype, as observed for TAMs. N1 neutrophils differentiate following TGFβ blockade and express immuno-activating cytokines and chemokines, low levels of Arg 1, and can kill cancer cells. On the opposite, N2 neutrophils are induced following exposure to high TGFβ levels [98], are characterized by expression of CXCR4, VEGF, and MMP9, and can inhibit CD8^+^ T cell function [99]. Of note, TANs have been linked to a poorer prognosis for patients with renal and pancreatic cancer; gastric, hepatocellular, colorectal, head and neck carcinomas, and melanoma [100, 101]. A few studies have highlighted the crosstalk between CAFs and TANs. For example, CAF-derived CXCL12/SDF1 and CXCR2 are involved in TANs recruitment within the TME and CAF-derived IL-6 stimulates signal transducer and activator of transcription 3 (STAT3) signaling pathway in TANs, potentially inducing immune tolerance through the expression of PD-L1 [102]. CAF-secreted TGFβ can also probably redirect TANs differentiation toward an N2 phenotype [98]. Furthermore, in hepatocellular carcinoma, CAF-derived CLCF1 increases CXCL6 and TGFβ secretion by tumor cells, which subsequently promotes TAN infiltration and polarization [103]. It seems that CAFs can also induce pro-tumorigenic neutrophil extracellular traps (NETs) formation in an amyloid β-dependent manner [104]. In a mouse breast tumor model, it was also shown that CAF-derived IL-33 facilitates lung metastasis by the recruitment of TANs [105]. Interestingly, the N2 polarization is also increased by vascular mimicry between CAF and cancer cells [106]. Finally, reciprocal crosstalk probably exists between CAFs and TANs. For example, neutrophil NETs can promote liver micro-metastasis in pancreatic ductal adenocarcinoma via the activation of CAFs [107] and TANs are capable to promote the differentiation of MSCs into CAFs [108]. It was also shown that the CAF marker PDPN interacts with the neutrophil protein CD177, with possible implications for CAF functions [109].

#### MCs and CAFs

MCs are tissue-resident sentinel cells that, upon activation, release a wide spectrum of chemokines and cytokines. MCs are mostly known for their role in an allergy but can also modulate tumor initiation and progression. Depending on their localization or cancer type, MCs exert dual effects on tumor progression [110]. As such, it seems that MCs display two subtypes, anti-tumorigenic MC1 and pro-tumorigenic MC2, which produce different mediators with opposite roles in tumorigenesis. In particular, MC1 produces IL-9, and histamine, which induces DC maturation and inhibits tumor growth in murine models. In contrast, MC2 produces a variety of angiogenic and metastatic substances, including VEGF, FGF, MMP9, TGFβ, and cytokines (IL-1β, IL-6, and IL-13) [111, 112]. Importantly, MCs can also alter the anti-tumor immune response. For example, the release of free adenosine [113] or IL-13 by MCs can respectively inhibit T cell function and promotes M2 polarization [114–116]. MCs can also favor the generation of highly suppressive MDSCs and Tregs in the TME [117, 118]. To date, research on the cooperation between MCs and CAFs in tumors is still in its infancy, with only a few studies addressing this question. For example, in odontogenic lesions that affect the jaw or neurofibroma, a large number CAFs and MCs in tumor islets are associated with the aggressiveness of the disease [119, 120]. In pancreatic tumors, stellate cells (a CAF precursor) can activate MCs which in turn enhance CAF proliferation by their secretion of IL-13 and tryptase. This process results in the formation of a fibrotic TME and ultimately suppresses the antitumor immune response [121]. Finally, in an *in vitro* three-dimensional (3D) microtissue model of prostate cancer, a recent study has revealed cooperation between MCs and CAFs, which enhances the transition from a benign to an anormal epithelia via a tryptase-dependent mechanism [122].

#### DCs and CAFs

In the TME, important antigen-presenting cell subpopulation, known as DCs, have a pivotal role in the activation of T cell-mediated, adaptive, anti-tumor immunity [123] and their global biology can be affected by the CAFs, even if in-depth mechanisms remain poorly understood. As a major source of TGFβ in the TME, CAFs can probably affect DC functions, in particular through the inhibition of MHC class II molecules, co-stimulatory molecules (CD40, CD80, and CD86), and cytokines (TNF-α, IFNγ, and IL-12) expression/secretion [124], which alter CD8^+^ cytotoxic T cell activation and Th1 polarization of CD4^+^ Th cell populations, and also promote the formation of CD4^+^ forkhead box protein P3 (FoxP3)^+^ Treg cells that potently inhibit the function of other T cells [125, 126]. Similarly, in hepatocellular carcinoma, CAFs have been described as a major source of IL-6 that affects DC functions through the activation of the STAT3 pathway leading to the generation of regulatory DCs, characterized by low expression of costimulatory molecules and high secretion of immune-suppressive cytokines, which impair T-cell proliferation and promote Tregs expansion [127]. Furthermore, CAF-produced IL-6 can also favor the emergence of pro-tumorigenic TAMs from monocytes at the expense of DCs [82]. Interestingly, in lung tumors, galectin1-driven secretion of TDO2 and IDO by CAFs promotes tryptophan degradation in kynurenines that inhibits DCs differentiation and functions [128]. In pancreatic tumors, the secretion of TNF-α and IL-1β by tumor cells promotes CAFs activation and their secretion of TSLP, which favor the generation of DCs with Th2-polarizing capabilities, associated with reduced patient survival [129]. In mouse esophageal squamous cell carcinoma, CAFs-secreted Wnt family member (WNT2) has been linked to suppression of the DC-initiated antitumor T-cell response via the suppressor of cytokine signaling 3 (SOCS3)/phosphorylated Janus kinase 2 (p-JAK2)/phosphorylated STAT3 (p-STAT3) signaling pathway. On the opposite, anti-WNT2 monoclonal antibodies (mAbs) can significantly restore T-cell responses and enhance the efficacy of anti-programmed cell death 1 (PD-1) therapy by increasing active DCs [130]. Furthermore, in ovarian cancers, CAFs can secrete wingless-type mouse mammary tumor virus integration site 16B (WNT16B) in response to DNA damage-associated treatment, which promotes the secretion of IL-10 and TGFβ by DCs [131]. Finally, as a major source of VEGF, CAFs might inhibit DC generation, maturation, and functions through this pathway [132].

#### NK cells and CAFs

CAFs can also alter the activity of NK cells, which are a major participant in the early immune response through their cytotoxic functions, and contribute to the adaptive immune response through their secretion of cytokines and the promotion of DC maturation. The detailed mechanisms of the complex relationship between CAFs and NK cells are still emerging and most likely involve multiple molecules. As such, TGFβ released in the TME by CAFs most likely plays an important role in the alteration of NK cell activation and cytotoxic activity [133], for example by reducing NK-activating receptor expression [134–136]. Furthermore, more direct evidence of the effect of CAFs on NK cells has been provided during the past few years. Independent studies involving melanoma, colorectal, and hepatocellular carcinoma-derived fibroblasts have shown that CAFs, through the secretion of PGE2 and IDO, can decrease the expression of several natural cytotoxicity receptors [NCRs, e.g., NKp30, NKp44 and NK receptor DNAX accessory molecule (DNAM)] at the NK cell surface, as well as perforin and granzyme B [137–139], leading to attenuated cytotoxic capabilities of NK cells. We also demonstrated that melanoma-associated CAFs decrease the sensitivity of melanoma tumor cells to NK cell-mediated killing through the secretion of MMPs which cleave MHC class I-related chain (MIC)-A and MIC-B [two ligands of NK group 2D (NKG2D)], at the surface of the tumor cells and consequently decrease both NKG2D-dependent cytotoxic activity of NK and their secretion of IFNγ [140]. In pancreatic ductal models, the high expression of the glutamatergic pre-synaptic protein netrin G1 (NetG1) in CAFs is also linked to their ability to inhibit NK cell-mediated killing of tumor cells [141]. Furthermore, in endometrial cancer, CAFs can decrease NK cells’ lytic potential through their downregulation of poliovirus receptor (PVR/CD155), a ligand of the activating DNAM-1/CD226 [142]. Finally, in the context of radiotherapy, CAFs isolated from non-small cell lung cancer inhibit NK cell activation and cytotoxic functions [143].

In summary, due to their secretion of cytokines, chemokines or other soluble factors, and possibly other mechanisms, CAFs shape the TME and favor the recruitment of innate immune cells and their acquisition of an immunosuppressive phenotype like M2 macrophages, N2 neutrophils, possibly MC2, but also affect DC functions or cytotoxic potential and cytokine production of NK cells.

### CAF-mediated interference with the adaptive anti-tumor immune response

CAFs also hamper the adaptive anti-tumor immune response at different levels, ultimately leading to the alteration of effector T cell functions in the TME (Figure 2). Of note, among FAP^HIGH^ CAF, the recent single cell analyses revealing the heterogeneity within this population mentioned earlier in this review have also strongly suggested that specific clusters, in particular those characterized by wound-healing signature, ECM accumulation, and TGFβ-signaling, are particularly associated with an immunosuppressive environment, at least in some tumor types [70, 144].

#### T lymphocytes and CAFs

As mentioned above, CAFs are an important source of TGFβ in the TME which acts on both CD8^+^ and CD4^+^ T cells [124, 126] and consequently hamper the antitumor T cell-dependent immune response and the response to immunotherapies. For example, in breast cancer, one of the cellular clusters identified among FAP^HIGH^ CAFs is characterized by TGFβ signaling and is linked to immunosuppression and resistance to immunotherapy [70]. Similarly, poor response to immunotherapies in the metastatic urothelial, lung, and colon cancer and melanoma have been linked to TGFβ signature in CAFs [145, 146]. Furthermore, in an ovarian cancer cohort, it has been shown that the key determinant of T cell exclusion is the up-regulation of TGFβ in the activated stromal compartment [147]. Mechanistically, TGFβ is known to have pleiotropic “bad” effects on the T cell-dependent immune response. This includes the alteration of effector CD8^+^ T cell survival through the inhibition of the pro-survival protein B cell lymphoma-2 (Bcl-2) expression [148], the reduction of CD8^+^ T cell cytolytic functions through the reduction of perforin, granzymes A and B, Fas ligand (CD95L) and IFNγ expression [149, 150], the reduction of CD8^+^ T cells infiltration [145], the alteration of the acquisition of effector function by memory CD8^+^ T cells [149, 151] or the promotion of Tregs recruitment and differentiation [152]. As such, TGFβ-secreting myCAF is very abundant in immune-excluded ovarian tumors [153], and αSMA^+^FAP^+^ CAFs from head and neck tumors have been shown to inhibit CD8^+^ T cell proliferation and to promote the recruitment of Tregs in a TGFβ-dependent manner [154]. Of note, it has been suggested that CAFs and Tregs enter into a reciprocal cross-talk via their mutual expression of TGFβ, increasing in parallel CAFs activation and Tregs activity [155].

Furthermore, CAFs are also an important source of cytokines and chemokines in the TME, with once again a potential pleiotropic effect on T cells. For example, in αSMA^+^FAP^+^ CAFs from head and neck tumors mentioned above, IL-6 secretion cooperates with TGFβ to inhibit CD8^+^ T cell proliferation and promote the recruitment of Tregs [154]. Similarly, in murine PDAC models, IL-6 depletion specifically in αSMA^+^ CAFs synergizes with anti-PD-1 immunotherapy to significantly improve the survival of tumor-bearing mice [156]. In breast cancer, CAF-derived IL-33 has been identified as a driver of the Th2-polarized immune response [25]. Furthermore, in lung and pancreatic tumors, the secretion of CXCL12/SDF1 by CAFs contributes to the exclusion of T cells from the cancer cell proximity [157, 158]. Similarly, in high-grade serous ovarian cancers, CAF-S1 increases the attraction, survival, and differentiation of Tregs via microRNA-141/200a (miR-141/200a)-dependent secretion of CXCL12β [61]. Similarly, recent scRNAseq analysis in breast cancer has also demonstrated that CXCL12 is highly secreted by iCAFs [70]. In the TME, CAFs-secreted CCL2, CCL5, CCL17, IL-1, IL-6, IL-13, and IL-26 can also promote a Th2 and Th17 CD4^+^ polarization, at the expense of anti-tumor Th1 response [33, 159–161]. Consequently, *in vivo* elimination of CAFs in a murine model of breast cancer using a vaccine targeting FAP can shift CD4^+^ T cell polarization from a Th2 to a Th1, increase expression of IL-2, increase CD8^+^ T cell functions, and hamper Tregs recruitment [161].

Of note, and as mentioned earlier in this review, the presence of CAFs in the TME profoundly affects the ECM through the deposition of several components (e.g., fibronectin or type I collagen) and proteolytic degradation of normal ECM structure in an MMPs-dependent manner. This remodeling has important consequences on both tumor behavior [23, 162–165] and the efficacy of the antitumor immune response [166]. Indeed, this modified ECM is presumed to restrict access of immune cells to cancer cells, serving as a physical barrier [166, 167]. The perfect example is PDAC, where fibrosis is extensive and the “scar-like” ECM acts as a barrier for cytotoxic T cell infiltration into tumor cell areas [168, 169]. This also occurs in other cancer types such as lung tumors, where T cells poorly migrate in dense ECM areas [158, 170]. Similarly, the presence of FAP^HIGH^ ECM-secreting CAFs has been linked to the exclusion of CD8^+^ T cells from the tumor and their accumulation in the collagen-rich peritumoral stroma [70]. Furthermore, in tumors with the accumulation of matrix proteins in the ECM, tumor tissues are often poorly oxygenated, resulting in the presence of areas with a low oxygen pressure called “hypoxic zones” [16, 171, 172]. Interestingly, several studies indicated that hypoxia is involved in the process of CAFs activation and in their functionality within the TME [173–177]. In parallel, in melanoma, our group recently provided evidence that hypoxia increases CAFs TGFβ, IL-6, IL-10, VEGF, and PD-L1 expression and/or secretion and demonstrates that hypoxic CAF exerts a more profound effect on T cell-mediated cytotoxicity than their normoxic counterpart [178].

In addition, CAFs can also impair T cell proliferation and effector functions through the metabolic reprogramming of the TME. In particular, the secretion by CAFs of IDO1 [179, 180], an immuno-regulatory enzyme that catabolizes tryptophan degradation [181, 182], or Arg 2, an enzyme involved in the deprivation of arginine in the TME [183], have a potentially important effect on T cells. In this regard, a poor clinical outcome for PDAC patients has been linked to the presence of CAFs expressing Arg 2 in hypoxic zones [184]. In addition, CAFs can use aerobic glycolysis as a source of energy, which results in the production of pyruvate and lactate that switch T cell polarization, reducing the percentage of Th1 CD4^+^ T cells and increasing Treg recruitment [185–187]. Furthermore, stromal cells from cervical tumors express high levels of CD39 and CD73, two molecules known to hydrolyze ATP, generating free adenosine, which possesses important immunosuppressive properties [188]. Similarly, FAP^HIGH^ CAFs from breast, colorectal and ovarian cancer express high levels of CD73, which potentially promotes immunosuppression in, at least in part, a Tregs-dependent manner [61, 189, 190]. Interestingly, it was also recently shown that CAFs upregulate CD39 expression on T cells, and in turn, T cells upregulate CD73 expression on CAFs [191]. CAFs highly express cyclooxygenase-2 (COX2) and are consequently a major source of PGE2 [192, 193], with important implications in the field of immunosuppression [194], especially by shifting the balance between Th1 and Th2 responses, by suppressing CD8^+^ T cell-mediated cytotoxic activity and by promoting Treg recruitment. As such, PDAC-derived CAFs strongly inhibited T-cell proliferation in a PGE2-dependent manner, and its inhibition by indomethacin, a non-steroidal anti-inflammatory molecule, partially restored the proliferative capacities of both CD4^+^ and CD8^+^ T-cells [195]. Finally, in breast cancer, NO secretion by FAP^HIGH^ PDPN^+^ CAFs can suppress T cell proliferation [196].

Finally, CAF can hamper T-cell-mediated immune response through many other miscellaneous mechanisms, many of them being under investigation or probably not yet elucidated. For example, it was suggested that CAFs can trigger cytotoxic T cell apoptosis via their expression of FasL/CD95L [197] and that CAFs secretion of galectins that have a high affinity for β-galactosides [198, 199], alters T cell functions [200–202].

#### MDSCs and CAFs

CAFs in the TME can also interfere with the adaptive immune response by facilitating the infiltration and generation of MDSCs, involved in the direct or indirect alteration of the T cell-mediated immune response through their secretion of several factors including Arg, iNOS, TGFβ, IL-10, PGE2 and IDO [203, 204]. In this regard, in pancreatic tumors, CAF-secreted IL-6 favors monocyte precursors differentiation towards an MDSC phenotype, in a STAT3-dependent manner [85, 205]. Similar results were obtained in esophageal squamous cell carcinoma, where CAF-secreted IL-6 and CAF-derived, exosome-packed, miR-21 promote MDSC differentiation via STAT3 signaling [206]. Furthermore, secretion of CXCL12/SDF1 by hepatic carcinoma-derived CAFs attracts monocytes into the tumor stroma and engages their differentiation into MDSCs in an IL-6- and STAT3-dependent manner [207]. MDSC-promoting factors (e.g., IL-6, VEGF, M-CSF, CXCL12, CCL2) can also be produced by pancreatic stellate cells, described as CAFs precursors [85]. Similar results were also obtained in murine liver tumor models, where FAP^+^ CAFs are a major source of CCL2 that enhances the recruitment of MDSCs and predicts poor prognosis of patients with intrahepatic cholangiocarcinoma [208]. In lung squamous cell carcinoma, CAFs have also been reported to promote peripheral CCR2^+^ monocyte migration via CCL2 and their reprogramming into MDSCs [209]. Another study described similar effects of CAF-secreted CXCL16 on monocytes in triple-negative breast cancers [210].

#### CAFs and immune checkpoints

Immune checkpoint receptors and ligands respectively expressed at the surface of T-cells and tumor cells, have clearly emerged as one of the main contributors to T-cell dysfunction within the TME. In particular, PD-L1 and PD-L2, two members of the B7 family of co-stimulatory/co-inhibitory molecules expressed by a large variety of cancer cells, engage their receptor PD-1 expressed on T-cells. This interaction strongly counteracts T cell receptor (TCR) signaling and CD28-co-stimulation [211], which result in the inhibition of T cell activation, proliferation, and functions. As such, PD-L1/PD-1 blocking antibodies now receive great attention in the field of tumor immunotherapies, especially in melanoma, lung, and renal cell carcinomas [212].

Very interestingly, several studies have now demonstrated that CAFs can express some of these immune checkpoint molecules. For example, CAFs from renal, colon, and lung cancers or melanoma can express programmed PD-L1, PD-L2, or CD276 (also known as B7-H3) [178, 197, 213–216], with potential participation in T cell exhaustion. Interestingly, the expression of some immune checkpoint ligands, especially OX40 ligand (OX40L)/CD242 and PD-L2, by FAP^+^ CAFs also allows their long-term interaction with Tregs, at least *in vitro* [61]. In parallel, CAFs can contribute to the expression of these immune checkpoints by other cell populations present within the TME, through their various production of cytokines or exosomes. As such, in pancreatic cancer, CAFs have been reported to increase the expression of PD-1, cytotoxic lymphocyte-associated antigen-4 (CTLA-4), lymphocyte-activation gene-3 (LAG-3), and T cell immunoglobulin and mucin-domain containing-3 (TIM-3), on both CD4^+^ and CD8^+^ T cell [195]. Similarly, in breast cancer, FAP^HIGH^ ECM-myCAF can recruit Tregs and increase PD-1 and CTLA-4 expression at their surface [70]. Moreover, CAF-secreted IL-6 can induce PD-L1 expression on TANs in a STAT3-dependent manner [102]. Importantly, CAF can also promote the expression of immune checkpoint ligands by tumor cells. For example, CAF-secreted CXCL5 was involved in the expression of PD-L1 on mouse melanoma and colorectal tumor cells in a phosphatidylinositol 3-kinase (PI3K)/AKT-dependent manner [217]. Similarly, in lung adenocarcinoma, CXCL2 secretion by αSMA^+^ CAFs increases PD-L1 expression by tumor cells [218] and αSMA^+^ CXCL5-secreting CAFs are positively correlated to PD-L1 expression by melanoma and colorectal carcinoma tumor cells [217]. Additionally, in human breast cancer, CAF-derived exosomes containing miR-92 decrease the expression of large tumor suppressor 2 (LATS2), and secondarily promotes the nuclear translocation of yes-associated protein 1 (YAP1) and its binding to the enhancer region of *PD-L1* to promote its transcription within tumor cells [219]. Nevertheless, it is crucial to emphasize that further studies are clearly needed to clarify both the mechanisms of CAF-induced immune checkpoint expression by the diverse population present in the TME and the influence of immune checkpoint ligand expression by CAF on the T cell-mediated anti-tumor immune response.

In summary, CAF can shape the adaptive antitumor immune response by switching CD4^+^ Th lymphocytes polarization from a Th1 to a Th2 phenotype, affecting Tregs and Th17 cells generation, affecting CD8^+^ T cell functions, modifying the ECM and T cell migration, by affecting MDSCs generation or through their effect on immune checkpoint receptors/ligands expression.

## CAFs targeting: a promising strategy to improve the efficacy of anti-tumor immune response and combined immunotherapies?

**Figure 3.**
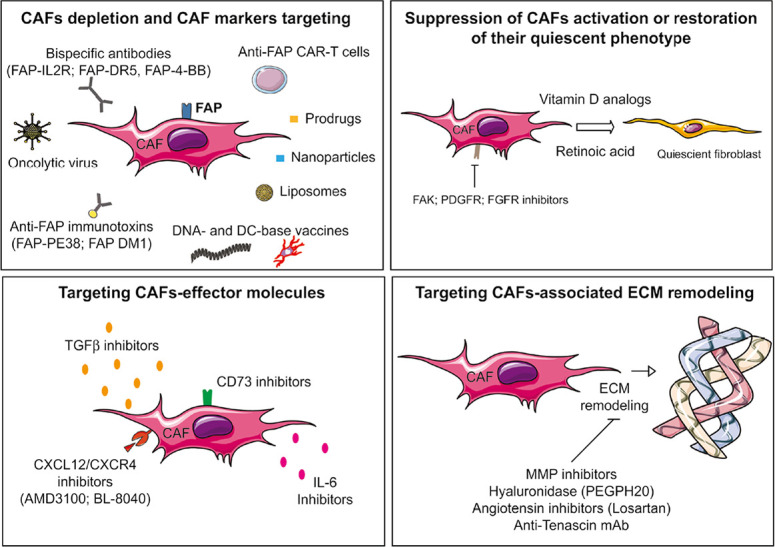
Schematic representation of the current main strategies to target CAFs. Several strategies are being explored in preclinical and/or clinical studies to target CAF-associated immunosuppression such as depletion of CAFs, restoration of their quiescent phenotype, targeting of CAFs-effector molecules or targeting of CAFs-associated ECM remodeling. FAK: focal adhesion kinase; FGFR: FGF receptor

Based on the capacities of CAFs to impair the anti-tumor immunity, and more generally exert pro-tumorigenic effects, the development of therapeutic strategies to target these cells in the TME is very seductive to improve the antitumor immune response and more generally may represent a great therapeutic advance in the fight against cancer. Several strategies are thus being explored in preclinical and/or clinical studies [220, 221] and mainly rely on depletion of CAFs, targeting of CAFs surface markers, restoration of their quiescent phenotype, targeting of CAFs-effector molecules, or targeting of CAFs-associated ECM remodeling [23] (Figure 3 and Table 1). Of note, it is also important to consider that the specificity of these therapeutic strategies is a real challenge. In other words, challenging research is needed for the development of anti-CAF therapies capable of specifically modulating CAFs activity without side effects on their normal counterparts, as normal fibroblasts can also be considered, under certain circumstances, as factors that limit tumor growth and invasiveness.

**Table 1.** Examples of clinical trials targeting CAFs

**Strategy**	**Approach**	**Indications**	**Combination**	**Trial ID**
CAF depletion	Anti Nectin-4 and FAP CAR T cells	Nectin4-positive advanced malignant solid tumors	-	NCT03932565
	FAP-IL-2R (R06874281)	Advanced or metastatic melanoma	Anti-PD-1	NCT03875079
		Unresectable advanced and/or metastatic renal cell carcinoma	Anti-PD-L1 ± anti-VEGF	NCT03063762
		Metastatic pancreatic ductal adenocarcinoma	Chemotherapy or anti-PD-L1	NCT03193190
		Breast cancer	Anti-Her2; anti-EGFR	NCT02627274
	FAP inhibitor (talabostat/BXCL701)	Advanced solid cancers	Anti-PD-1	NCT04171219
Suppression of CAF activation	Vitamin D	Cervical/uterine cancer	Radiation or anti-PD-1	NCT03192059
		Metastatic pancreatic ductal adenocarcinoma	Chemotherapy or anti-PD-1	NCT02754726
Targeting CAF-effector molecules	CXCR4 antagonist motixafortide (BL-8040)	Metastatic pancreatic cancer	Anti-PD-1	NCT02826486
	CD73 blockade	Advanced solid tumors	Anti-PD-1	NCT02754141
Targeting CAF-induced ECM remodeling	Pegylated recombinant hyaluronidase (PEGPH20)	Pancreatic cancer	Chemotherapy	NCT01839487
		Gastric, gastroesophageal, or esophageal cancer	Chemotherapy or anti-PD-L1	NCT03281369

CAR: chimeric antigen-receptor; EGFR: EGF receptor

### Strategies to deplete CAFs or to redirect local immune response against CAF surface markers

To date, CAF-depleting therapies have been mainly focused on strategies targeting cell surface markers. Based on a pioneer study demonstrating that FAP genetic depletion in mouse models causes rapid necrosis of both Lewis lung tumor cells and stromal cells in an IFNγ, TNF-α and CD8^+^ T cells-dependent manner [222, 223], many direct CAFs depletion strategies have been developed to target this marker [224, 225], such as vaccination approaches or CAR T cells. For example, an oral DNA vaccine targeting FAP has successfully demonstrated its ability to induce CD8^+^ T cell-mediated killing of CAF and to suppress primary tumor cell growth and metastasis of colon and breast murine carcinoma [226]. Murine LL2 (lung cancer), B16F10 (melanoma), and CT26 (colon cancer) tumor cells modified to express FAP, used as a whole-tumor cell vaccine, can induce antitumor immunity against both tumor cells and CAFs, with a notable enhancement of CD8^+^ T lymphocytes infiltration and a decrease of immunosuppressive cell accumulation within the TME [227]. Similarly, in murine melanoma models, the vaccination-based depletion of FAP^+^ stromal cells has been linked to the reduction of immunosuppressive cell frequencies and functions, resulting in a robust CD8^+^ T cell response and prolonged survival of melanoma-bearing mice [223]. More recently, a synthetic consensus sequence approach to provide MHC class II help was used to develop a FAP DNA vaccine, which was shown to synergize with other tumor antigen-specific DNA vaccines to enhance CD8^+^ and CD4^+^ antitumor immunity [228]. A FAP vaccine using a modified vaccinia Ankara vector combined with cyclophosphamide also significantly enhanced anti-tumor immune response decreased Tregs infiltration, and prolonged the survival of 4T1 tumor-bearing mice [229]. Furthermore, a DC vaccine that encodes an A20-specific short hairpin RNA (shRNA) to enhance DC function, targets FAP and the tumor antigen tyrosinase-related protein 2 (TRP-2), has demonstrated its ability to enhance tumor infiltration of CD8^+^ T cells and to induce robust FAP- and TRP-2-specific T-cell responses in a B16 melanoma model [230]. More innovative approaches such as the fusion of DCs with FAP^+^ CAFs have been developed, for example in H22 mouse hepatoma models, and can efficiently stimulate T cell-mediated immune response *in vitro* and inhibit the growth of H22 xenografts *in vivo* [231]. Similarly, in colon, melanoma, lung, and breast cancer models, exosome-like nanovesicles derived from *FAP*-engineered tumor cells have been used as a vaccine that inhibits tumor growth by a cytotoxic T lymphocyte (CTL)-mediated immune response against both tumor cells and FAP^+^ CAFs [232]. Furthermore, the development of CAR T cells targeting FAP has also shown promising results in murine models [233–235] and in malignant pleural mesothelioma patient-derived xenograft (PDX) models [236] and are now in clinical trials (see [237] for review). For example, a clinical trial using a fourth-generation CAR T targeting Nectin-4 and FAP in advanced malignant solid tumors (NCT03932565) is ongoing. Furthermore, recent studies also investigated the use of a bispecific antibody (R06874281/Simlukafusp alfa) which binds to FAP on CAF and IL-2 receptor on immune cells [238]. This approach was designed to stimulate antibody-dependent or T cell-dependent cellular cytotoxicity against CAFs, increase the pool of CD8^+^ T and NK cells immune effectors, and reduce Tregs activity [238]. Of note, given its promising preclinical results, several clinical trials are ongoing using R06874281 (e.g., NCT03875079; NCT03193190; NCT02627274; NCT03063762). Similarly, an optimized tetravalent FAP-DR5 bispecific antibody (RG7386) was developed [239], as well as a bispecific antibody targeting FAP and 4-1BB/CD137 [240, 241]. Finally, other FAP-targeting approaches have been developed. For example, the FAP inhibitor talabostat/BXCL701 [242] has been used in a phase II trial as a single agent for patients with metastatic colorectal cancer [242] or in association with cisplatin in melanoma [243] and is currently tested in association with anti-PD-1 therapy in advance solid cancers (NCT04171219). Finally, CAF depleting strategies also include immunotoxin targeting FAP, such as FAP-PE38 [244] or FAP-DM1 [245], FAP targeting oncolytic adenovirus [234, 246], liposomes [247], prodrugs [248, 249], nanoparticles [250]; nanocarriers [251], light-activated nanohyperthermia [252, 253], small molecules such as ABT-263 [254] or anti-FAP antibodies labeled with ^131^Iodine [255].

In summary, CAFs depleting strategies have been mainly focused on FAP, even if a clinical trial targeting PDGFR, another CAF marker, is ongoing using dasatinib [256]. Nevertheless, it should be noted that, in addition to CAFs, FAP can be expressed by cells present in several tissues, including multipotent bone marrow stem cells or skeletal muscles, with potential side effects of the strategies targeting FAP, as suggested [257], highlighting caution against its use as a universal target. This last point also suggests that more highly selective markers are probably required to improve the precision of CAF depletion-based therapies. In this regard, targeting CD10 and G protein-coupled receptor 77 (GPR77), two markers for a specific CAF subset that correlates with chemoresistance and poor survival in multiple cohorts of breast and lung cancer patients, could be an effective therapeutic strategy, as suggested [63].

### Suppression of CAFs activation or restoration of their quiescent phenotype

Another strategy to restrain CAFs function within the TME relies on the normalization of their quiescent state. To date, this approach mainly uses retinoic acid (a metabolite of vitamin A) or vitamin D [37], even if other approaches exist or will certainly emerge. Indeed, as already mentioned, vitamin A or D deficiency can promote CAF activation [35–37]. Consequently, it was hypothesized that targeting this pathway may enable CAFs conversion back to the normal quiescent state. In this regard, in 2D and 3D PDAC models, all-trans retinoic acid (ATRA) treatment reverts CAFs to a quiescent state together with a reduction of proliferation and increased apoptosis of surrounding pancreatic cancer cells [36]. Similarly, treatment with calcipotriol, a vitamin D receptor ligand, reprogram the tumor stroma to a more quiescent state, which improves gemcitabine delivery in PDAC tumors and ultimately enhances antitumor activity compared to chemotherapy alone [258]. Consequently, several clinical trials are ongoing to evaluate the clinical efficacy of vitamin D analogs, in combination with other treatments, especially immunotherapies. For example, a phase II study is ongoing to evaluate the combination of vitamin D with PD-1 inhibitors and radiation in a patient with advanced and refractory cervical cancer, endometrial carcinoma, or uterine sarcoma (NCT03192059), and treatment with vitamin D in association with chemo- or anti-PD-1-therapies are currently evaluated for patients with pancreatic cancer (NCT02754726). Nevertheless, a recent study in PDAC demonstrated that calcipotriol, a vitamin D3 analog, reduces the tumor supportive activity of CAFs, but at the same time decreases T cell effector functions, which highlights the needed caution with this approach [259]. Finally, targeted therapies that could modulate pathways involved in CAF activation have been developed. For example, the FAK pathway is potentially an important target since it promotes the emergence of a fibrotic and inflammatory TME and is essential for CAF development. As such, FAK inhibitors have demonstrated a synergistic effect with PD-1 inhibitors in PDAC models [260]. Similarly, targeting the Hedgehog signaling [261] pathway has been also considered [262]. Several indirect potential targets are also currently explored. For example, pharmacological inhibition of nicotinamide adenine dinucleotide phosphate oxidase 4 (NOX4) using GKT137831, a small organic molecule of the pyrazolopyridine dione chemical class, prevents and reverses ROS-dependent myofibroblast activation [263]. Other potential targets are for example PDGFR or FGFR [264].

### Targeting CAFs-effector molecules

Because depleting CAFs from the TME is still challenging, targeting the CAF secretome to attenuate their immunosuppressive role in the TME is also an interesting strategy. However, it is important to note that this approach is less specific since the immunomodulatory factors expressed and secreted by CAFs are also expressed and secreted by other cell populations within the TME and by the tumor cells. The importance of TGFβ in the activation of CAFs and its crucial role in their immunosuppressive capabilities makes this cytokine an obvious target. For example, artemisinin inactivates CAFs by the suppression of TGFβ signaling in breast cancer [265] and tranilast (Rizaben), a known suppressor of fibroblast proliferation and TGFβ secretion, has demonstrated a synergistic effect with a DC-based vaccine in C57BL/6 mice bearing syngeneic E-G7 lymphoma, LLC1 Lewis lung cancer or B16F1 melanoma [266]. Consequently, multiple preclinical and clinical trials using TGFβ-targeting drugs (including neutralizing antibodies, ligand traps, small-molecule kinase inhibitors, or antisense oligonucleotides) alone or in combination with immunotherapies or other treatments are ongoing, even if the current results are, at least partly, disappointing [267, 268]. Another potential target is CXCL12. In this regard, a crucial study has demonstrated that targeting CXCL12 from FAP^+^ CAFs with plerixafor (AMD3100) synergizes with anti-PD-L1 treatment in pancreatic cancer [157]. The immunosuppressive axis driven by CAFs in a CXCL12-CXCR4-dependent manner is also targeted by the CXCR4 antagonist motixafortide (BL-8040) in combination with anti-PD-1 antibodies in phase II clinical trial for patients with pancreatic cancer (NCT02826486). Since CAFs also secrete high levels of IL-6, which negatively affect NK and T cell functions, IL-6 or IL-6 receptor (IL-6R)-targeting agents [269] could also be useful to interfere with CAFs immunosuppressive activity. Finally, as CAFs have been identified as CD73 highly expressing cells [189], blocking this ectonucleotidase is probably a way of choice to hamper CAFs immunosuppressive effects, in synergy with anti-PD-1 or anti-CTLA-4 antibodies [270–272], cancer vaccines [273] or CAR T cells [274]. Accordingly, several clinical trials using CD73 blocking are ongoing in combination with immune checkpoint blockade, targeted- or chemo-therapies (e.g., NCT02754141).

### Targeting CAFs-associated ECM remodeling

As mentioned earlier, CAF-induced ECM remodeling is an important future that affects immune effector cell recruitment to cancer cell areas [166, 167]. Consequently, targeting the ECM remodeling is a potential therapeutic option to hamper CAFs-mediated immunosuppression. One potential CAF-produced target currently explored is hyaluronan (HA). HA is a large aminoglycan and a key ECM component involved in stromal fibrosis [275]. Mechanistically, HA-enriched TME promotes tumor vasculature compression in a collagen-dependent manner, resulting in tumor hypoxia, and also blocks the delivery of peripheral immune cells or drugs from blood vessels to tumors [276]. Consequently, HA-targeting approaches have been developed, such as PEGPH20, a pegylated recombinant hyaluronidase. PEGPH20 facilitates tumor reoxygenation and the intra-tumoral penetration of chemotherapeutic agents in preclinical models [276–278] and displays therapeutic benefit in association with gemcitabine for patients with advanced pancreatic cancer (NCT01839487) [279]. However, more recent data have demonstrated the poor clinical benefit of this treatment in association with paclitaxel/gemcitabine in patients with HA^HIGH^ metastatic pancreatic ductal adenocarcinoma [280]. PEGPH20 is also currently tested for gastric or esophageal cancers (NCT03281369). Moreover, the angiotensin II inhibitor losartan also displays the ability to decrease collagen and HA production by inhibiting TGFβ, connective tissue growth factor (CTGF), and endothelin-1 (ET-1) profibrotic signals, and consequently improves drug and oxygen delivery to tumors, thereby potentiating chemotherapy and reducing hypoxia in breast and pancreatic cancer models [281]. Nevertheless, to date, the effect of PEGPH20 or Losartan on immune effector cell infiltration within tumors has never been addressed.

TN-C, a glycoprotein expressed in the ECM of several tissues and overexpressed in a variety of cancer tissues is also a potential target [282]. Indeed, recent studies have demonstrated that CAFs express TN-C in many tumors [283] and several antibodies have been engineered to target this protein. For example, F16 and P12 antibodies specific to the alternatively spliced domains of the large isoform of TN-C [284], have been fused with IL-2 to promote CD45^+^ immune cell recruitment and tested in a xenograft model of human breast cancer [285]. Interestingly, it was also shown that antibody-based inhibition of TN-C in autophagy-deficient breast cancer cells improves their CTL-mediated killing and the efficacy of a single anti-PD-1/PD-L1 treatment [286].

Other potential CAF-produced targets are also actually explored such as MMPs, which greatly influence ECM degradation. However, despite promising preclinical results supporting the use of MMPs inhibitor for cancer treatment, the obtained clinical data have been disappointing [286]. Nevertheless, as more specific MMPs inhibitors are now developed, MMPs targeting will be probably reconsidered, especially to target CAF-secreted MMPs to improve immune responses. In this regard, we have shown *in vitro* that the inhibition of MMPs secreted by melanoma-associated CAFs improves the NK-mediated killing of melanoma tumor cells [140].

## Conclusions

Despite their abundance in the TME, fibroblasts have been ignored over decades, but are now considered a major player in tumor initiation and progression. Meanwhile, an increasing amount of research has revealed their heterogeneity in terms of origins and subsets, which also reflects their pleiotropic functions in tumor growth and the variety of chemokines, cytokines, and other factors secreted within the TME. Additionally, their function in the alteration of the antitumor immune response is now widely recognized, thanks to the extensive efforts which made it possible to grasp their secretome and its complex immunosuppressive network that affect both innate and adaptive immune system. Furthermore, CAFs are now considered targets that can be manipulated through therapeutic intervention, as demonstrated by the numerous clinical trials involving CAF-targeting agents used as monotherapy or in combination with existing treatments. These approaches are also expected to enhance immune effector cell infiltration and cytotoxic functions within the tumor, and to enhance the efficacy of current immunotherapy approaches. Nevertheless, multiple challenges are still ahead, such as the definition of CAFs more specific markers, the precise definition of the different CAF subpopulation functions and their localization during tumor progression, and finally the development of targeting agents that are specific enough to spare normal stromal cells in healthy tissues. Furthermore, it is also important to note that some CAFs subsets exert tumor-inhibiting effects, it is therefore conceivable that, under certain circumstances or tumor tissues, these cells may act as both heroes and villains [287], making this field even more challenging.

## Abbreviations

3D: three-dimensional
Arg: arginase
CAFs: cancer-associated fibroblasts
CAR: chimeric antigen-receptor
CAV1: caveolin-1
CCL2: C-C motif chemokine ligand 2
Chi3L1: chitinase-3-like-1
CLCF1: cardiotrophin-like cytokine factor 1
CTLA-4: cytotoxic lymphocyte-associated antigen-4
CXCL12: chemokine C-X-C motif ligand 12
CXCR2: C-X-C chemokine receptor 2
DCs: dendritic cells
ECM: extracellular matrix
FAK: focal adhesion kinase
FAP: fibroblast-activation protein
FGF: fibroblast growth factor
FGFR: fibroblast growth factor receptor
FSP1: fibroblast-specific protein-1
HA: hyaluronan
iCAF: inflammatory cancer-associated fibroblast
IDO: indoleamine-2,3-dioxygenase
IFNγ: interferon-γ
IL-1β: interleukin-1β
iNOS: inducible nitric oxide synthase
MCP-1: monocyte chemoattractant protein-1
MCs: mast cells
M-CSF: macrophage colony-stimulating factor
MDSCs: myeloid-derived suppressor cells
MHC: major histocompatibility complex
miR-141/200a: microRNA-141/200a
MMPs: matrix metallo-proteinases
MSCs: mesenchymal stem cells
myCAF: myofibroblastic cancer-associated fibroblast
NK: natural killer
NO: nitric oxide
PD-1: programmed cell death 1
PDAC: pancreatic ductal carcinoma
PDGFRs: platelet-derived growth factor receptors
PD-L1: programmed death ligand 1
PDPN: podoplanin
PGE2: prostaglandin E2
ROS: reactive oxygen species
SDF1: stromal cell-derived factor-1
STAT3: signal transducer and activator of transcription 3
TAMs: tumor-associated macrophages
TANs: tumor-associated neutrophils
TGFβ: transforming growth factor-β
Th: T-helper
TME: tumor microenvironment
TN-C: tenascin-C
TNF: tumor necrosis factor
Tregs: regulatory T cells
VEGF: vascular endothelial growth factor
αSMA: α-smooth muscle actin

## Declarations

### Copyright

© The Author(s) 2022.

## References

[1] Balkwill FR Capasso M Hagemann T. The tumor microenvironment at a glance. J Cell Sci. 2012;125:5591–6. 10.1242/jcs.116392 23420197

[2] Quail DF Joyce JA. Microenvironmental regulation of tumor progression and metastasis. Nat Med. 2013;19:1423–37. 10.1038/nm.3394 24202395 PMC3954707

[3] Hanahan D. Hallmarks of cancer: new dimensions. Cancer Discov. 2022;12:31–46. 10.1158/2159-8290.CD-21-1059 35022204

[4] Chen F Zhuang X Lin L Yu P Wang Y Shi Y New horizons in tumor microenvironment biology: challenges and opportunities. BMC Med. 2015;13:45. 10.1186/s12916-015-0278-7 25857315 PMC4350882

[5] Baghban R Roshangar L Jahanban-Esfahlan R Seidi K Ebrahimi-Kalan A Jaymand M Tumor microenvironment complexity and therapeutic implications at a glance. Cell Commun Signal. 2020;18:59. 10.1186/s12964-020-0530-4 32264958 PMC7140346

[6] Binnewies M Roberts EW Kersten K Chan V Fearon DF Merad M Understanding the tumor immune microenvironment (TIME) for effective therapy. Nat Med. 2018;24:541–50. 10.1038/s41591-018-0014-x 29686425 PMC5998822

[7] Gajewski TF Schreiber H Fu YX. Innate and adaptive immune cells in the tumor microenvironment. Nat Immunol. 2013;14:1014–22. 10.1038/ni.2703 24048123 PMC4118725

[8] Li X Yang Y Huang Q Deng Y Guo F Wang G Crosstalk between the tumor microenvironment and cancer cells: a promising predictive biomarker for immune checkpoint inhibitors. Front Cell Dev Biol. 2021;9:738373. 10.3389/fcell.2021.738373 34692696 PMC8529050

[9] Labani-Motlagh A Ashja-Mahdavi M Loskog A. The tumor microenvironment: a milieu hindering and obstructing antitumor immune responses. Front Immunol. 2020;11:940. 10.3389/fimmu.2020.00940 32499786 PMC7243284

[10] Cirri P Chiarugi P. Cancer associated fibroblasts: the dark side of the coin. Am J Cancer Res. 2011;1:482–97. 21984967 PMC3186047

[11] Kalluri R. The biology and function of fibroblasts in cancer. Nat Rev Cancer. 2016;16:582–98. 10.1038/nrc.2016.73 27550820

[12] Madar S Goldstein I Rotter V. ‘Cancer associated fibroblasts’--more than meets the eye. Trends Mol Med. 2013;19:447–53. 10.1016/j.molmed.2013.05.004 23769623

[13] Valcz G Sipos F Tulassay Z Molnar B Yagi Y. Importance of carcinoma-associated fibroblast-derived proteins in clinical oncology. J Clin Pathol. 2014;67:1026–31. 10.1136/jclinpath-2014-202561 25135950

[14] Fearon DT. The carcinoma-associated fibroblast expressing fibroblast activation protein and escape from immune surveillance. Cancer Immunol Res. 2014;2:187–93. 10.1158/2326-6066.CIR-14-0002 24778314

[15] Harper J Sainson RC. Regulation of the anti-tumour immune response by cancer-associated fibroblasts. Semin Cancer Biol. 2014;25:69–77. 10.1016/j.semcancer.2013.12.005 24406209

[16] Jiang H Hegde S DeNardo DG. Tumor-associated fibrosis as a regulator of tumor immunity and response to immunotherapy. Cancer Immunol Immunother. 2017;66:1037–48. 10.1007/s00262-017-2003-1 28451791 PMC5603233

[17] Ziani L Chouaib S Thiery J. Alteration of the antitumor immune response by cancer-associated fibroblasts. Front Immunol. 2018;9:414. 10.3389/fimmu.2018.00414 29545811 PMC5837994

[18] Mao X Xu J Wang W Liang C Hua J Liu J Crosstalk between cancer-associated fibroblasts and immune cells in the tumor microenvironment: new findings and future perspectives. Mol Cancer. 2021;20:131. 10.1186/s12943-021-01428-1 34635121 PMC8504100

[19] Mhaidly R Mechta-Grigoriou F. Role of cancer-associated fibroblast subpopulations in immune infiltration, as a new means of treatment in cancer. Immunol Rev. 2021;302:259–72. 10.1111/imr.12978 34013544 PMC8360036

[20] Desbois M Wang Y. Cancer-associated fibroblasts: key players in shaping the tumor immune microenvironment. Immunol Rev. 2021;302:241–58. 10.1111/imr.12982 34075584

[21] Darby IA Laverdet B Bonte F Desmouliere A. Fibroblasts and myofibroblasts in wound healing. Clin Cosmet Investig Dermatol. 2014;7:301–11. 10.2147/CCID.S50046 25395868 PMC4226391

[22] Micallef L Vedrenne N Billet F Coulomb B Darby IA Desmouliere A. The myofibroblast, multiple origins for major roles in normal and pathological tissue repair. Fibrogenesis Tissue Repair. 2012;5:S5. 10.1186/1755-1536-5-S1-S5 23259712 PMC3368789

[23] Gascard P Tlsty TD. Carcinoma-associated fibroblasts: orchestrating the composition of malignancy. Genes Dev. 2016;30:1002–19. 10.1101/gad.279737.116 27151975 PMC4863733

[24] Tomasek JJ Gabbiani G Hinz B Chaponnier C Brown RA. Myofibroblasts and mechano-regulation of connective tissue remodelling. Nat Rev Mol Cell Biol. 2002;3:349–63. 10.1038/nrm809 11988769

[25] Sahai E Astsaturov I Cukierman E DeNardo DG Egeblad M Evans RM A framework for advancing our understanding of cancer-associated fibroblasts. Nat Rev Cancer. 2020;20:174–86. 10.1038/s41568-019-0238-1 31980749 PMC7046529

[26] Kojima Y Acar A Eaton EN Mellody KT Scheel C Ben-Porath I Autocrine TGF-beta and stromal cell-derived factor-1 (SDF-1) signaling drives the evolution of tumor-promoting mammary stromal myofibroblasts. Proc Natl Acad Sci U S A. 2010;107:20009–14. 10.1073/pnas.1013805107 21041659 PMC2993333

[27] Vicent S Sayles LC Vaka D Khatri P Gevaert O Chen R Cross-species functional analysis of cancer-associated fibroblasts identifies a critical role for CLCF1 and IL-6 in non-small cell lung cancer *in vivo*. Cancer Res. 2012;72:5744–56. 10.1158/0008-5472.CAN-12-1097 22962265 PMC3856949

[28] Öhlund D Elyada E Tuveson D. Fibroblast heterogeneity in the cancer wound. J Exp Med. 2014;211:1503–23. 10.1084/jem.20140692 25071162 PMC4113948

[29] Bronzert DA Pantazis P Antoniades HN Kasid A Davidson N Dickson RB Synthesis and secretion of platelet-derived growth factor by human breast cancer cell lines. Proc Natl Acad Sci U S A. 1987;84:5763–7. 10.1073/pnas.84.16.5763 3039506 PMC298943

[30] Elenbaas B Weinberg RA. Heterotypic signaling between epithelial tumor cells and fibroblasts in carcinoma formation. Exp Cell Res. 2001;264:169–84. 10.1006/excr.2000.5133 11237532

[31] Kuzet SE Gaggioli C. Fibroblast activation in cancer: when seed fertilizes soil. Cell Tissue Res. 2016;365:607–19. 10.1007/s00441-016-2467-x 27474009

[32] Löhr M Schmidt C Ringel J Kluth M Müller P Nizze H Transforming growth factor-beta1 induces desmoplasia in an experimental model of human pancreatic carcinoma. Cancer Res. 2001;61:550–5. 11212248

[33] Erez N Truitt M Olson P Arron ST Hanahan D. Cancer-associated fibroblasts are activated in incipient neoplasia to orchestrate tumor-promoting inflammation in an NF-kappaB-dependent manner. Cancer Cell. 2010;17:135–47. Erratum in: Cancer Cell. 2010;17:523. 10.1016/j.ccr.2009.12.041 20138012

[34] Mazzocca A Dituri F Lupo L Quaranta M Antonaci S Giannelli G. Tumor-secreted lysophostatidic acid accelerates hepatocellular carcinoma progression by promoting differentiation of peritumoral fibroblasts in myofibroblasts. Hepatology. 2011;54:920–30. 10.1002/hep.24485 21674557

[35] Ferrer-Mayorga G Gómez-López G Barbáchano A Fernández-Barral A Peña C Pisano DG Vitamin D receptor expression and associated gene signature in tumour stromal fibroblasts predict clinical outcome in colorectal cancer. Gut. 2017;66:1449–62. 10.1136/gutjnl-2015-310977 27053631 PMC5530491

[36] Froeling FE Feig C Chelala C Dobson R Mein CE Tuveson DA Retinoic acid-induced pancreatic stellate cell quiescence reduces paracrine Wnt-β-catenin signaling to slow tumor progression. Gastroenterology. 2011;141:1486–97.e14. 10.1053/j.gastro.2011.06.047 21704588

[37] Shany S Sigal-Batikoff I Lamprecht S. Vitamin D and myofibroblasts in fibrosis and cancer: at cross-purposes with TGF-β/SMAD signaling. Anticancer Res. 2016;36:6225–34. 10.21873/anticanres.11216 27919940

[38] Zeisberg EM Potenta S Xie L Zeisberg M Kalluri R. Discovery of endothelial to mesenchymal transition as a source for carcinoma-associated fibroblasts. Cancer Res. 2007;67:10123–8. 10.1158/0008-5472.CAN-07-3127 17974953

[39] Yeon JH Jeong HE Seo H Cho S Kim K Na D Cancer-derived exosomes trigger endothelial to mesenchymal transition followed by the induction of cancer-associated fibroblasts. Acta Biomater. 2018;76:146–53. 10.1016/j.actbio.2018.07.001 30078422

[40] Hosaka K Yang Y Seki T Fischer C Dubey O Fredlund E Pericyte-fibroblast transition promotes tumor growth and metastasis. Proc Natl Acad Sci U S A. 2016;113:E5618–27. 10.1073/pnas.1608384113 27608497 PMC5035870

[41] Jotzu C Alt E Welte G Li J Hennessy BT Devarajan E Adipose tissue-derived stem cells differentiate into carcinoma-associated fibroblast-like cells under the influence of tumor-derived factors. Anal Cell Pathol (Amst). 2010;33:61–79. 10.3233/ACP-CLO-2010-0535 20978328 PMC4605656

[42] Kidd S Spaeth E Watson K Burks J Lu H Klopp A Origins of the tumor microenvironment: quantitative assessment of adipose-derived and bone marrow-derived stroma. PLoS One. 2012;7:e30563. 10.1371/journal.pone.0030563 22363446 PMC3282707

[43] Bochet L Lehuédé C Dauvillier S Wang YY Dirat B Laurent V Adipocyte-derived fibroblasts promote tumor progression and contribute to the desmoplastic reaction in breast cancer. Cancer Res. 2013;73:5657–68. 10.1158/0008-5472.CAN-13-0530 23903958

[44] Öhlund D Handly-Santana A Biffi G Elyada E Almeida AS Ponz-Sarvise M Distinct populations of inflammatory fibroblasts and myofibroblasts in pancreatic cancer. J Exp Med. 2017;214:579–96. 10.1084/jem.20162024 28232471 PMC5339682

[45] Okabe H Hayashi H Nakagawa S Imai K Nitta H Arima K Inducible factors for cancer-associated fibroblasts in liver cancer *versus* myofibroblasts in inflammatory liver disease. Histol Histopathol. 2016;31:141–8. 10.14670/HH-11-668 26398776

[46] Direkze NC Alison MR. Bone marrow and tumour stroma: an intimate relationship. Hematol Oncol. 2006;24:189–95. 10.1002/hon.788 16795113

[47] Direkze NC Hodivala-Dilke K Jeffery R Hunt T Poulsom R Oukrif D Bone marrow contribution to tumor-associated myofibroblasts and fibroblasts. Cancer Res. 2004;64:8492–5. 10.1158/0008-5472.CAN-04-1708 15574751

[48] Ishii G Sangai T Oda T Aoyagi Y Hasebe T Kanomata N Bone-marrow-derived myofibroblasts contribute to the cancer-induced stromal reaction. Biochem Biophys Res Commun. 2003;309:232–40. 10.1016/S0006-291X(03)01544-4 12943687

[49] Mishra PJ Mishra PJ Humeniuk R Medina DJ Alexe G Mesirov JP Carcinoma-associated fibroblast-like differentiation of human mesenchymal stem cells. Cancer Res. 2008;68:4331–9. 10.1158/0008-5472.CAN-08-0943 18519693 PMC2725025

[50] Raz Y Cohen N Shani O Bell RE Novitskiy SV Abramovitz L Bone marrow-derived fibroblasts are a functionally distinct stromal cell population in breast cancer. J Exp Med. 2018;215:3075–93. 10.1084/jem.20180818 30470719 PMC6279405

[51] Werner S Lützkendorf J Müller T Müller LP Posern G. MRTF-A controls myofibroblastic differentiation of human multipotent stromal cells and their tumour-supporting function in xenograft models. Sci Rep. 2019;9:11725. 10.1038/s41598-019-48142-z 31409840 PMC6692381

[52] Corsa CA Brenot A Grither WR Van Hove S Loza AJ Zhang K The action of discoidin domain receptor 2 in basal tumor cells and stromal cancer-associated fibroblasts is critical for breast cancer metastasis. Cell Rep. 2016;15:2510–23. 10.1016/j.celrep.2016.05.033 27264173 PMC4909540

[53] De Wever O Nguyen QD Van Hoorde L Bracke M Bruyneel E Gespach C Tenascin-C and SF/HGF produced by myofibroblasts *in vitro* provide convergent pro-invasive signals to human colon cancer cells through RhoA and Rac. FASEB J. 2004;18:1016–8. 10.1096/fj.03-1110fje 15059978

[54] Kelly T Huang Y Simms AE Mazur A. Fibroblast activation protein-alpha: a key modulator of the microenvironment in multiple pathologies. Int Rev Cell Mol Biol. 2012;297:83–116. 10.1016/B978-0-12-394308-8.00003-0 22608558

[55] Park JE Lenter MC Zimmermann RN Garin-Chesa P Old LJ Rettig WJ. Fibroblast activation protein, a dual specificity serine protease expressed in reactive human tumor stromal fibroblasts. J Biol Chem. 1999;274:36505–12. 10.1074/jbc.274.51.36505 10593948

[56] Strutz F Okada H Lo CW Danoff T Carone RL Tomaszewski JE Identification and characterization of a fibroblast marker: FSP1. J Cell Biol. 1995;130:393–405. 10.1083/jcb.130.2.393 7615639 PMC2199940

[57] Sugimoto H Mundel TM Kieran MW Kalluri R. Identification of fibroblast heterogeneity in the tumor microenvironment. Cancer Biol Ther. 2006;5:1640–6. 10.4161/cbt.5.12.3354 17106243

[58] True LD Zhang H Ye M Huang CY Nelson PS von Haller PD CD90/THY1 is overexpressed in prostate cancer-associated fibroblasts and could serve as a cancer biomarker. Mod Pathol. 2010;23:1346–56. 10.1038/modpathol.2010.122 20562849 PMC2948633

[59] Yurugi Y Wakahara M Matsuoka Y Sakabe T Kubouchi Y Haruki T Podoplanin expression in cancer-associated fibroblasts predicts poor prognosis in patients with squamous cell carcinoma of the lung. Anticancer Res. 2017;37:207–13. 10.21873/anticanres.11308 28011493

[60] Mercier I Casimiro MC Wang C Rosenberg AL Quong J Minkeu A Human breast cancer-associated fibroblasts (CAFs) show caveolin-1 downregulation and RB tumor suppressor functional inactivation: implications for the response to hormonal therapy. Cancer Biol Ther. 2008;7:1212–25. 10.4161/cbt.7.8.6220 18458534 PMC6688494

[61] Costa A Kieffer Y Scholer-Dahirel A Pelon F Bourachot B Cardon M Fibroblast heterogeneity and immunosuppressive environment in human breast cancer. Cancer Cell. 2018;33:463–79.e10. 10.1016/j.ccell.2018.01.011 29455927

[62] Givel AM Kieffer Y Scholer-Dahirel A Sirven P Cardon M Pelon F miR200-regulated CXCL12beta promotes fibroblast heterogeneity and immunosuppression in ovarian cancers. Nat Commun. 2018;9:1056. 10.1038/s41467-018-03348-z 29535360 PMC5849633

[63] Su S Chen J Yao H Liu J Yu S Lao L CD10^+^ GPR77^+^ cancer-associated fibroblasts promote cancer formation and chemoresistance by sustaining cancer stemness. Cell. 2018;172:841–56.e16. 10.1016/j.cell.2018.01.009 29395328

[64] Pelon F Bourachot B Kieffer Y Magagna I Mermet-Meillon F Bonnet I Cancer-associated fibroblast heterogeneity in axillary lymph nodes drives metastases in breast cancer through complementary mechanisms. Nat Commun. 2020;11:404. 10.1038/s41467-019-14134-w 31964880 PMC6972713

[65] Peltier A Seban RD Buvat I Bidard FC Mechta-Grigoriou F. Fibroblast heterogeneity in solid tumors: from single cell analysis to whole-body imaging. Semin Cancer Biol. 2022;86:262–72. 10.1016/j.semcancer.2022.04.008 35489628

[66] Elyada E Bolisetty M Laise P Flynn WF Courtois ET Burkhart RA Cross-species single-cell analysis of pancreatic ductal adenocarcinoma reveals antigen-presenting cancer-associated fibroblasts. Cancer Discov. 2019;9:1102–23. 10.1158/2159-8290.CD-19-0094 31197017 PMC6727976

[67] Neuzillet C Tijeras-Raballand A Ragulan C Cros J Patil Y Martinet M Inter- and intra-tumoural heterogeneity in cancer-associated fibroblasts of human pancreatic ductal adenocarcinoma. J Pathol. 2019;248:51–65. 10.1002/path.5224 30575030 PMC6492001

[68] Li H Courtois ET Sengupta D Tan Y Chen KH Goh JJL Reference component analysis of single-cell transcriptomes elucidates cellular heterogeneity in human colorectal tumors. Nat Genet. 2017;49:708–18. Erratum in: Nat Genet. 2018;50:1754. 10.1038/ng.3818 28319088

[69] Lambrechts D Wauters E Boeckx B Aibar S Nittner D Burton O Phenotype molding of stromal cells in the lung tumor microenvironment. Nat Med. 2018;24:1277–89. 10.1038/s41591-018-0096-5 29988129

[70] Kieffer Y Hocine HR Gentric G Pelon F Bernard C Bourachot B Single-cell analysis reveals fibroblast clusters linked to immunotherapy resistance in cancer. Cancer Discov. 2020;10:1330–51. 10.1158/2159-8290.CD-19-1384 32434947

[71] Bartoschek M Oskolkov N Bocci M Lövrot J Larsson C Sommarin M Spatially and functionally distinct subclasses of breast cancer-associated fibroblasts revealed by single cell RNA sequencing. Nat Commun. 2018;9:5150. 10.1038/s41467-018-07582-3 30514914 PMC6279758

[72] Chen Y McAndrews KM Kalluri R. Clinical and therapeutic relevance of cancer-associated fibroblasts. Nat Rev Clin Oncol. 2021;18:792–804. 10.1038/s41571-021-00546-5 34489603 PMC8791784

[73] Kanzaki R Pietras K. Heterogeneity of cancer-associated fibroblasts: opportunities for precision medicine. Cancer Sci. 2020;111:2708–17. 10.1111/cas.14537 32573845 PMC7419037

[74] Louault K Li RR DeClerck YA. Cancer-associated fibroblasts: understanding their heterogeneity. Cancers (Basel). 2020;12:3108. 10.3390/cancers12113108 33114328 PMC7690906

[75] Simon T Salhia B. Cancer-associated fibroblast subpopulations with diverse and dynamic roles in the tumor microenvironment. Mol Cancer Res. 2022;20:183–92. 10.1158/1541-7786.MCR-21-0282 34670861 PMC9306405

[76] Paraiso KH Smalley KS. Fibroblast-mediated drug resistance in cancer. Biochem Pharmacol. 2013;85:1033–41. 10.1016/j.bcp.2013.01.018 23376122

[77] Hinshaw DC Shevde LA. The tumor microenvironment innately modulates cancer progression. Cancer Res. 2019;79:4557–66. 10.1158/0008-5472.CAN-18-3962 31350295 PMC6744958

[78] Mantovani A Marchesi F Malesci A Laghi L Allavena P. Tumour-associated macrophages as treatment targets in oncology. Nat Rev Clin Oncol. 2017;14:399–416. 10.1038/nrclinonc.2016.217 28117416 PMC5480600

[79] Fujii N Shomori K Shiomi T Nakabayashi M Takeda C Ryoke K Cancer-associated fibroblasts and CD163-positive macrophages in oral squamous cell carcinoma: their clinicopathological and prognostic significance. J Oral Pathol Med. 2012;41:444–51. 10.1111/j.1600-0714.2012.01127.x 22296275

[80] Herrera M Herrera A Domínguez G Silva J García V García JM Cancer-associated fibroblast and M2 macrophage markers together predict outcome in colorectal cancer patients. Cancer Sci. 2013;104:437–44. 10.1111/cas.12096 23298232 PMC7657228

[81] Kuen J Darowski D Kluge T Majety M. Pancreatic cancer cell/fibroblast co-culture induces M2 like macrophages that influence therapeutic response in a 3D model. PLoS One. 2017;12:e0182039. 10.1371/journal.pone.0182039 28750018 PMC5531481

[82] Chomarat P Banchereau J Davoust J Palucka AK. IL-6 switches the differentiation of monocytes from dendritic cells to macrophages. Nat Immunol. 2000;1:510–4. 10.1038/82763 11101873

[83] Comito G Giannoni E Segura CP Barcellos-de-Souza P Raspollini MR Baroni G Cancer-associated fibroblasts and M2-polarized macrophages synergize during prostate carcinoma progression. Oncogene. 2014;33:2423–31. 10.1038/onc.2013.191 23728338

[84] Laoui D Van Overmeire E De Baetselier P Van Ginderachter JA Raes G. Functional relationship between tumor-associated macrophages and macrophage colony-stimulating factor as contributors to cancer progression. Front Immunol. 2014;5:489. 10.3389/fimmu.2014.00489 25339957 PMC4188035

[85] Mace TA Ameen Z Collins A Wojcik S Mair M Young GS Pancreatic cancer-associated stellate cells promote differentiation of myeloid-derived suppressor cells in a STAT3-dependent manner. Cancer Res. 2013;73:3007–18. 10.1158/0008-5472.CAN-12-4601 23514705 PMC3785672

[86] Takahashi H Sakakura K Kudo T Toyoda M Kaira K Oyama T Cancer-associated fibroblasts promote an immunosuppressive microenvironment through the induction and accumulation of protumoral macrophages. Oncotarget. 2017;8:8633–47. 10.18632/oncotarget.14374 28052009 PMC5352428

[87] Zhang J Chen L Xiao M Wang C Qin Z. FSP1^+^ fibroblasts promote skin carcinogenesis by maintaining MCP-1-mediated macrophage infiltration and chronic inflammation. Am J Pathol. 2011;178:382–90. 10.1016/j.ajpath.2010.11.017 21224075 PMC3070559

[88] Cohen N Shani O Raz Y Sharon Y Hoffman D Abramovitz L Fibroblasts drive an immunosuppressive and growth-promoting microenvironment in breast cancer via secretion of chitinase 3-like 1. Oncogene. 2017;36:4457–68. 10.1038/onc.2017.65 28368410 PMC5507301

[89] Miyake M Hori S Morizawa Y Tatsumi Y Nakai Y Anai S CXCL1-mediated interaction of cancer cells with tumor-associated macrophages and cancer-associated fibroblasts promotes tumor progression in human bladder cancer. Neoplasia. 2016;18:636–46. Erratum in: Neoplasia. 2017;19:250–1. 10.1016/j.neo.2016.12.012 27690238 PMC5043399

[90] Zhou J Wang XH Zhao YX Chen C Xu XY Sun Q Cancer-associated fibroblasts correlate with tumor-associated macrophages infiltration and lymphatic metastasis in triple negative breast cancer patients. J Cancer. 2018;9:4635–41. 10.7150/jca.28583 30588247 PMC6299377

[91] Gok Yavuz B Gunaydin G Gedik ME Kosemehmetoglu K Karakoc D Ozgur F Cancer associated fibroblasts sculpt tumour microenvironment by recruiting monocytes and inducing immunosuppressive PD-1^+^ TAMs. Sci Rep. 2019;9:3172. 10.1038/s41598-019-39553-z 30816272 PMC6395633

[92] Zhang R Qi F Zhao F Li G Shao S Zhang X Cancer-associated fibroblasts enhance tumor-associated macrophages enrichment and suppress NK cells function in colorectal cancer. Cell Death Dis. 2019;10:273. 10.1038/s41419-019-1435-2 30894509 PMC6426970

[93] Mazur A Holthoff E Vadali S Kelly T Post SR. Cleavage of type I collagen by fibroblast activation protein-alpha enhances class A scavenger receptor mediated macrophage adhesion. PLoS One. 2016;11:e0150287. 10.1371/journal.pone.0150287 26934296 PMC4774960

[94] Zhang Q Chai S Wang W Wan C Zhang F Li Y Macrophages activate mesenchymal stem cells to acquire cancer-associated fibroblast-like features resulting in gastric epithelial cell lesions and malignant transformation *in vitro*. Oncol Lett. 2019;17:747–56. 10.3892/ol.2018.9703 30655826 PMC6313054

[95] Hashimoto O Yoshida M Koma Y Yanai T Hasegawa D Kosaka Y Collaboration of cancer-associated fibroblasts and tumour-associated macrophages for neuroblastoma development. J Pathol. 2016;240:211–23. 10.1002/path.4769 27425378 PMC5095779

[96] Masucci MT Minopoli M Carriero MV. Tumor associated neutrophils. Their role in tumorigenesis, metastasis, prognosis and therapy. Front Oncol. 2019;9:1146. 10.3389/fonc.2019.01146 31799175 PMC6874146

[97] Shaul ME Fridlender ZG. Tumour-associated neutrophils in patients with cancer. Nat Rev Clin Oncol. 2019;16:601–20. 10.1038/s41571-019-0222-4 31160735

[98] Fridlender ZG Sun J Kim S Kapoor V Cheng G Ling L Polarization of tumor-associated neutrophil phenotype by TGF-beta: “N1” *versus* “N2” TAN. Cancer Cell. 2009;16:183–94. 10.1016/j.ccr.2009.06.017 19732719 PMC2754404

[99] Leliefeld PH Koenderman L Pillay J. How neutrophils shape adaptive immune responses. Front Immunol. 2015;6:471. 10.3389/fimmu.2015.00471 26441976 PMC4568410

[100] Ocana A Nieto-Jiménez C Pandiella A Templeton AJ. Neutrophils in cancer: prognostic role and therapeutic strategies. Mol Cancer. 2017;16:137. 10.1186/s12943-017-0707-7 28810877 PMC5558711

[101] Wang Y Zhai J Zhang T Han S Zhang Y Yao X Tumor-associated neutrophils can predict lymph node metastasis in early gastric cancer. Front Oncol. 2020;10:570113. 10.3389/fonc.2020.570113 33072602 PMC7537418

[102] Cheng Y Li H Deng Y Tai Y Zeng K Zhang Y Cancer-associated fibroblasts induce PDL1+ neutrophils through the IL6-STAT3 pathway that foster immune suppression in hepatocellular carcinoma. Cell Death Dis. 2018;9:422. 10.1038/s41419-018-0458-4 29556041 PMC5859264

[103] Song M He J Pan QZ Yang J Zhao J Zhang YJ Cancer-associated fibroblast-mediated cellular crosstalk supports hepatocellular carcinoma progression. Hepatology. 2021;73:1717–35. 10.1002/hep.31792 33682185

[104] Munir H Jones JO Janowitz T Hoffmann M Euler M Martins CP Stromal-driven and amyloid beta-dependent induction of neutrophil extracellular traps modulates tumor growth. Nat Commun. 2021;12:683. 10.1038/s41467-021-20982-2 33514748 PMC7846803

[105] Shani O Vorobyov T Monteran L Lavie D Cohen N Raz Y Fibroblast-derived IL33 facilitates breast cancer metastasis by modifying the immune microenvironment and driving type 2 immunity. Cancer Res. 2020;80:5317–29. 10.1158/0008-5472.CAN-20-2116 33023944 PMC7611300

[106] Tsai YM Wu KL Liu YW Chang WA Huang YC Chang CY Cooperation between cancer and fibroblasts in vascular mimicry and N2-type neutrophil recruitment *via* Notch2-Jagged1 interaction in lung cancer. Front Oncol. 2021;11:696931. 10.3389/fonc.2021.696931 34485133 PMC8415962

[107] Takesue S Ohuchida K Shinkawa T Otsubo Y Matsumoto S Sagara A Neutrophil extracellular traps promote liver micrometastasis in pancreatic ductal adenocarcinoma via the activation of cancerassociated fibroblasts. Int J Oncol. 2020;56:596–605. 10.3892/ijo.2019.4951 31894273

[108] Zhu Q Zhang X Zhang L Li W Wu H Yuan X The IL-6-STAT3 axis mediates a reciprocal crosstalk between cancer-derived mesenchymal stem cells and neutrophils to synergistically prompt gastric cancer progression. Cell Death Dis. 2014;5:e1295. 10.1038/cddis.2014.263 24946088 PMC4611735

[109] Astarita JL Keerthivasan S Husain B Şenbabaoğlu Y Verschueren E Gierke S The neutrophil protein CD177 is a novel PDPN receptor that regulates human cancer-associated fibroblast physiology. PLoS One. 2021;16:e0260800. 10.1371/journal.pone.0260800 34879110 PMC8654239

[110] Derakhshani A Vahidian F Alihasanzadeh M Mokhtarzadeh A Lotfi Nezhad P Baradaran B. Mast cells: a double-edged sword in cancer. Immunol Lett. 2019;209:28–35. 10.1016/j.imlet.2019.03.011 30905824

[111] Soongsathitanon J Jamjuntra P Sumransub N Yangngam S De la Fuente M Landskron G Crosstalk between tumor-infiltrating immune cells and cancer-associated fibroblasts in tumor growth and immunosuppression of breast cancer. J Immunol Res. 2021;2021:8840066. 10.1155/2021/8840066 34337083 PMC8294979

[112] Varricchi G Galdiero MR Loffredo S Marone G Iannone R Marone G Are mast cells MASTers in cancer? Front Immunol. 2017;8:424. 10.3389/fimmu.2017.00424 28446910 PMC5388770

[113] Marquardt DL Gruber HE Wasserman SI. Adenosine release from stimulated mast cells. Proc Natl Acad Sci U S A. 1984;81:6192–6. 10.1073/pnas.81.19.6192 6435127 PMC391886

[114] Allard B Beavis PA Darcy PK Stagg J. Immunosuppressive activities of adenosine in cancer. Curr Opin Pharmacol. 2016;29:7–16. 10.1016/j.coph.2016.04.001 27209048

[115] Martinez-Nunez RT Louafi F Sanchez-Elsner T. The interleukin 13 (IL-13) pathway in human macrophages is modulated by microRNA-155 via direct targeting of interleukin 13 receptor alpha1 (IL13Ralpha1). J Biol Chem. 2011;286:1786–94. 10.1074/jbc.M110.169367 21097505 PMC3023473

[116] Lichterman JN Reddy SM. Mast cells: a new frontier for cancer immunotherapy. Cells. 2021;10:1270. 10.3390/cells10061270 34063789 PMC8223777

[117] Danelli L Frossi B Pucillo CE. Mast cell/MDSC a liaison immunosuppressive for tumor microenvironment. Oncoimmunology. 2015;4:e1001232. 10.1080/2162402X.2014.1001232 26137400 PMC4485753

[118] Yang Z Zhang B Li D Lv M Huang C Shen GX Mast cells mobilize myeloid-derived suppressor cells and Treg cells in tumor microenvironment via IL-17 pathway in murine hepatocarcinoma model. PLoS One. 2010;5:e8922. 10.1371/journal.pone.0008922 20111717 PMC2811741

[119] Pereira JDS de Oliveira Nóbrega FJ Vasconcelos RG de Souza Martins Câmara AC de Souza LB Queiroz LMG. Myofibroblasts and mast cells: influences on biological behavior of odontogenic lesions. Ann Diagn Pathol. 2018;34:66–71. 10.1016/j.anndiagpath.2014.09.003 29661731

[120] Yang FC Chen S Clegg T Li X Morgan T Estwick SA *Nf1*+/- mast cells induce neurofibroma like phenotypes through secreted TGF-beta signaling. Hum Mol Genet. 2006;15:2421–37. 10.1093/hmg/ddl165 16835260 PMC3024714

[121] Ma Y Hwang RF Logsdon CD Ullrich SE. Dynamic mast cell-stromal cell interactions promote growth of pancreatic cancer. Cancer Res. 2013;73:3927–37. 10.1158/0008-5472.CAN-12-4479 23633481 PMC3702652

[122] Pereira BA Lister NL Hashimoto K Teng L Flandes-Iparraguirre M Eder Aet al.; Melbourne Urological Research Alliance (MURAL). Tissue engineered human prostate microtissues reveal key role of mast cell-derived tryptase in potentiating cancer-associated fibroblast (CAF)-induced morphometric transition *in vitro*. Biomaterials. 2019;197:72–85. 10.1016/j.biomaterials.2018.12.030 30641266

[123] Mildner A Jung S. Development and function of dendritic cell subsets. Immunity. 2014;40:642–56. 10.1016/j.immuni.2014.04.016 24837101

[124] Flavell RA Sanjabi S Wrzesinski SH Licona-Limón P. The polarization of immune cells in the tumour environment by TGFbeta. Nat Rev Immunol. 2010;10:554–67. 10.1038/nri2808 20616810 PMC3885992

[125] Raker VK Domogalla MP Steinbrink K. Tolerogenic dendritic cells for regulatory T cell induction in man. Front Immunol. 2015;6:569. 10.3389/fimmu.2015.00569 26617604 PMC4638142

[126] Travis MA Sheppard D. TGF-beta activation and function in immunity. Annu Rev Immunol. 2014;32:51–82. 10.1146/annurev-immunol-032713-120257 24313777 PMC4010192

[127] Cheng JT Deng YN Yi HM Wang GY Fu BS Chen WJ Hepatic carcinoma-associated fibroblasts induce IDO-producing regulatory dendritic cells through IL-6-mediated STAT3 activation. Oncogenesis. 2016;5:e198. 10.1038/oncsis.2016.7 26900950 PMC5154347

[128] Hsu YL Hung JY Chiang SY Jian SF Wu CY Lin YS Lung cancer-derived galectin-1 contributes to cancer associated fibroblast-mediated cancer progression and immune suppression through TDO2/kynurenine axis. Oncotarget. 2016;7:27584–98. 10.18632/oncotarget.8488 27050278 PMC5053673

[129] De Monte L Reni M Tassi E Clavenna D Papa I Recalde H Intratumor T helper type 2 cell infiltrate correlates with cancer-associated fibroblast thymic stromal lymphopoietin production and reduced survival in pancreatic cancer. J Exp Med. 2011;208:469–78. 10.1084/jem.20101876 21339327 PMC3058573

[130] Huang TX Tan XY Huang HS Li YT Liu BL Liu KS Targeting cancer-associated fibroblast-secreted WNT2 restores dendritic cell-mediated antitumour immunity. Gut. 2022;71:333–44. 10.1136/gutjnl-2020-322924 33692094 PMC8762012

[131] Shen CC Kang YH Zhao M He Y Cui DD Fu YY WNT16B from ovarian fibroblasts induces differentiation of regulatory T cells through beta-catenin signal in dendritic cells. Int J Mol Sci. 2014;15:12928–39. 10.3390/ijms150712928 25050785 PMC4139882

[132] Yang J Yan J Liu B. Targeting VEGF/VEGFR to modulate antitumor immunity. Front Immunol. 2018;9:978. 10.3389/fimmu.2018.00978 29774034 PMC5943566

[133] Wu Y Tian Z Wei H. Developmental and functional control of natural killer cells by cytokines. Front Immunol. 2017;8:930. 10.3389/fimmu.2017.00930 28824650 PMC5543290

[134] Castriconi R Cantoni C Della Chiesa M Vitale M Marcenaro E Conte R Transforming growth factor beta 1 inhibits expression of NKp30 and NKG2D receptors: consequences for the NK-mediated killing of dendritic cells. Proc Natl Acad Sci U S A. 2003;100:4120–5. 10.1073/pnas.0730640100 12646700 PMC153058

[135] Donatelli SS Zhou JM Gilvary DL Eksioglu EA Chen X Cress WD TGF-beta-inducible microRNA-183 silences tumor-associated natural killer cells. Proc Natl Acad Sci U S A. 2014;111:4203–8. 10.1073/pnas.1319269111 24586048 PMC3964044

[136] Park YP Choi SC Kiesler P Gil-Krzewska A Borrego F Weck J Complex regulation of human NKG2D-DAP10 cell surface expression: opposing roles of the gammac cytokines and TGF-β1. Blood. 2011;118:3019–27. 10.1182/blood-2011-04-346825 21816829 PMC3291493

[137] Balsamo M Scordamaglia F Pietra G Manzini C Cantoni C Boitano M Melanoma-associated fibroblasts modulate NK cell phenotype and antitumor cytotoxicity. Proc Natl Acad Sci U S A. 2009;106:20847–52. 10.1073/pnas.0906481106 19934056 PMC2791633

[138] Li T Yang Y Hua X Wang G Liu W Jia C Hepatocellular carcinoma-associated fibroblasts trigger NK cell dysfunction via PGE2 and IDO. Cancer Lett. 2012;318:154–61. 10.1016/j.canlet.2011.12.020 22182446

[139] Li T Yi S Liu W Jia C Wang G Hua X Colorectal carcinoma-derived fibroblasts modulate natural killer cell phenotype and antitumor cytotoxicity. Med Oncol. 2013;30:663. 10.1007/s12032-013-0663-z 23873014

[140] Ziani L Safta-Saadoun TB Gourbeix J Cavalcanti A Robert C Favre G Melanoma-associated fibroblasts decrease tumor cell susceptibility to NK cell-mediated killing through matrix-metalloproteinases secretion. Oncotarget. 2017;8:19780–94. 10.18632/oncotarget.15540 28423623 PMC5386721

[141] Francescone R Barbosa Vendramini-Costa D Franco-Barraza J Wagner J Muir A Lau AN Netrin G1 promotes pancreatic tumorigenesis through cancer-associated fibroblast-driven nutritional support and immunosuppression. Cancer Discov. 2021;11:446–79. 10.1158/2159-8290.CD-20-0775 33127842 PMC7858242

[142] Inoue T Adachi K Kawana K Taguchi A Nagamatsu T Fujimoto A Cancer-associated fibroblast suppresses killing activity of natural killer cells through downregulation of poliovirus receptor (PVR/CD155), a ligand of activating NK receptor. Int J Oncol. 2016;49:1297–304. 10.3892/ijo.2016.3631 27499237 PMC5021244

[143] Yang N Lode K Berzaghi R Islam A Martinez-Zubiaurre I Hellevik T. Irradiated tumor fibroblasts avoid immune recognition and retain immunosuppressive functions over natural killer cells. Front Immunol. 2021;11:602530. 10.3389/fimmu.2020.602530 33584669 PMC7874190

[144] Dominguez CX Müller S Keerthivasan S Koeppen H Hung J Gierke S Single-cell RNA sequencing reveals stromal evolution into LRRC15^+^ myofibroblasts as a determinant of patient response to cancer immunotherapy. Cancer Discov. 2020;10:232–53. 10.1158/2159-8290.CD-19-0644 31699795

[145] Mariathasan S Turley SJ Nickles D Castiglioni A Yuen K Wang Y TGFβ attenuates tumour response to PD-L1 blockade by contributing to exclusion of T cells. Nature. 2018;554:544–8. 10.1038/nature25501 29443960 PMC6028240

[146] Tauriello DVF Palomo-Ponce S Stork D Berenguer-Llergo A Badia-Ramentol J Iglesias M TGFβ drives immune evasion in genetically reconstituted colon cancer metastasis. Nature. 2018;554:538–43. 10.1038/nature25492 29443964

[147] Desbois M Udyavar AR Ryner L Kozlowski C Guan Y Dürrbaum M Integrated digital pathology and transcriptome analysis identifies molecular mediators of T-cell exclusion in ovarian cancer. Nat Commun. 2020;11:5583. 10.1038/s41467-020-19408-2 33149148 PMC7642433

[148] Sanjabi S Mosaheb MM Flavell RA. Opposing effects of TGF-beta and IL-15 cytokines control the number of short-lived effector CD8^+^ T cells. Immunity. 2009;31:131–44. 10.1016/j.immuni.2009.04.020 19604492 PMC2765785

[149] Ahmadzadeh M Rosenberg SA. TGF-beta 1 attenuates the acquisition and expression of effector function by tumor antigen-specific human memory CD8 T cells. J Immunol. 2005;174:5215–23. 10.4049/jimmunol.174.9.5215 15843517 PMC2562293

[150] Thomas DA Massagué J. TGF-beta directly targets cytotoxic T cell functions during tumor evasion of immune surveillance. Cancer Cell. 2005;8:369–80. 10.1016/j.ccr.2005.10.012 16286245

[151] Broderick L Bankert RB. Membrane-associated TGF-beta1 inhibits human memory T cell signaling in malignant and nonmalignant inflammatory microenvironments. J Immunol. 2006;177:3082–8. 10.4049/jimmunol.177.5.3082 16920945

[152] Chen W Jin W Hardegen N Lei KJ Li L Marinos N Conversion of peripheral CD4^+^CD25^–^ naive T cells to CD4^+^CD25^+^ regulatory T cells by TGF-beta induction of transcription factor Foxp3. J Exp Med. 2003;198:1875–86. 10.1084/jem.20030152 14676299 PMC2194145

[153] Hornburg M Desbois M Lu S Guan Y Lo AA Kaufman S Single-cell dissection of cellular components and interactions shaping the tumor immune phenotypes in ovarian cancer. Cancer Cell. 2021;39:928–44.e6. 10.1016/j.ccell.2021.04.004 33961783

[154] Takahashi H Sakakura K Kawabata-Iwakawa R Rokudai S Toyoda M Nishiyama M Immunosuppressive activity of cancer-associated fibroblasts in head and neck squamous cell carcinoma. Cancer Immunol Immunother. 2015;64:1407–17. 10.1007/s00262-015-1742-0 26201938 PMC11029788

[155] Kinoshita T Ishii G Hiraoka N Hirayama S Yamauchi C Aokage K Forkhead box P3 regulatory T cells coexisting with cancer associated fibroblasts are correlated with a poor outcome in lung adenocarcinoma. Cancer Sci. 2013;104:409–15. 10.1111/cas.12099 23305175 PMC7657221

[156] McAndrews KM Chen Y Darpolor JK Zheng X Yang S Carstens JL Identification of functional heterogeneity of carcinoma-associated fibroblasts with distinct IL6-mediated therapy resistance in pancreatic cancer. Cancer Discov. 2022;12:1580–97. 10.1158/2159-8290.CD-20-1484 35348629 PMC9399904

[157] Feig C Jones JO Kraman M Wells RJ Deonarine A Chan DS Targeting CXCL12 from FAP-expressing carcinoma-associated fibroblasts synergizes with anti-PD-L1 immunotherapy in pancreatic cancer. Proc Natl Acad Sci U S A. 2013;110:20212–7. 10.1073/pnas.1320318110 24277834 PMC3864274

[158] Salmon H Donnadieu E. Within tumors, interactions between T cells and tumor cells are impeded by the extracellular matrix. Oncoimmunology. 2012;1:992–4. 10.4161/onci.20239 23162783 PMC3489771

[159] Barnas JL Simpson-Abelson MR Brooks SP Kelleher RJ Jr Bankert RB. Reciprocal functional modulation of the activation of T lymphocytes and fibroblasts derived from human solid tumors. J Immunol. 2010;185:2681–92. 10.4049/jimmunol.1000896 20686130

[160] Haniffa MA Wang XN Holtick U Rae M Isaacs JD Dickinson AM Adult human fibroblasts are potent immunoregulatory cells and functionally equivalent to mesenchymal stem cells. J Immunol. 2007;179:1595–604. 10.4049/jimmunol.179.3.1595 17641026

[161] Liao D Luo Y Markowitz D Xiang R Reisfeld RA. Cancer associated fibroblasts promote tumor growth and metastasis by modulating the tumor immune microenvironment in a 4T1 murine breast cancer model. PLoS One. 2009;4:e7965. 10.1371/journal.pone.0007965 19956757 PMC2775953

[162] Fullár A Dudás J Oláh L Hollósi P Papp Z Sobel G Remodeling of extracellular matrix by normal and tumor-associated fibroblasts promotes cervical cancer progression. BMC Cancer. 2015;15:256. 10.1186/s12885-015-1272-3 25885552 PMC4409756

[163] Pickup MW Mouw JK Weaver VM. The extracellular matrix modulates the hallmarks of cancer. EMBO Rep. 2014;15:1243–53. 10.15252/embr.201439246 25381661 PMC4264927

[164] Winkler J Abisoye-Ogunniyan A Metcalf KJ Werb Z. Concepts of extracellular matrix remodelling in tumour progression and metastasis. Nat Commun. 2020;11:5120. 10.1038/s41467-020-18794-x 33037194 PMC7547708

[165] Henke E Nandigama R Ergün S. Extracellular matrix in the tumor microenvironment and its impact on cancer therapy. Front Mol Biosci. 2020;6:160. 10.3389/fmolb.2019.00160 32118030 PMC7025524

[166] Sorokin L. The impact of the extracellular matrix on inflammation. Nat Rev Immunol. 2010;10:712–23. 10.1038/nri2852 20865019

[167] Joyce JA Fearon DT. T cell exclusion, immune privilege, and the tumor microenvironment. Science. 2015;348:74–80. 10.1126/science.aaa6204 25838376

[168] Hartmann N Giese NA Giese T Poschke I Offringa R Werner J Prevailing role of contact guidance in intrastromal T-cell trapping in human pancreatic cancer. Clin Cancer Res. 2014;20:3422–33. 10.1158/1078-0432.CCR-13-2972 24763614

[169] Jiang H Hegde S Knolhoff BL Zhu Y Herndon JM Meyer MA Targeting focal adhesion kinase renders pancreatic cancers responsive to checkpoint immunotherapy. Nat Med. 2016;22:851–60. 10.1038/nm.4123 27376576 PMC4935930

[170] Salmon H Franciszkiewicz K Damotte D Dieu-Nosjean MC Validire P Trautmann A Matrix architecture defines the preferential localization and migration of T cells into the stroma of human lung tumors. J Clin Invest. 2012;122:899–910. 10.1172/JCI45817 22293174 PMC3287213

[171] Higgins DF Kimura K Bernhardt WM Shrimanker N Akai Y Hohenstein B Hypoxia promotes fibrogenesis *in vivo* via HIF-1 stimulation of epithelial-to-mesenchymal transition. J Clin Invest. 2007;117:3810–20. 10.1172/JCI30487 18037992 PMC2082142

[172] Moon JO Welch TP Gonzalez FJ Copple BL. Reduced liver fibrosis in hypoxia-inducible factor-1alpha-deficient mice. Am J Physiol Gastrointest Liver Physiol. 2009;296:G582–92. 10.1152/ajpgi.90368.2008 19136383 PMC2660171

[173] Comito G Giannoni E Di Gennaro P Segura CP Gerlini G Chiarugi P. Stromal fibroblasts synergize with hypoxic oxidative stress to enhance melanoma aggressiveness. Cancer Lett. 2012;324:31–41. 10.1016/j.canlet.2012.04.025 22659468

[174] Kugeratski FG Atkinson SJ Neilson LJ Lilla S Knight JRP Serneels J Hypoxic cancer-associated fibroblasts increase NCBP2-AS2/HIAR to promote endothelial sprouting through enhanced VEGF signaling. Sci Signal. 2019;12:eaan8247. 10.1126/scisignal.aan8247 30723174 PMC6794160

[175] Tang YA Chen YF Bao Y Mahara S Yatim SMJM Oguz G Hypoxic tumor microenvironment activates GLI2 via HIF-1alpha and TGF-beta2 to promote chemoresistance in colorectal cancer. Proc Natl Acad Sci U S A. 2018;115:E5990–9. 10.1073/pnas.1801348115 29891662 PMC6042102

[176] Tejchman A Lamerant-Fayel N Jacquinet JC Bielawska-Pohl A Mleczko-Sanecka K Grillon C Tumor hypoxia modulates podoplanin/CCL21 interactions in CCR7+ NK cell recruitment and CCR7+ tumor cell mobilization. Oncotarget. 2017;8:31876–87. 10.18632/oncotarget.16311 28416768 PMC5458255

[177] Giaccia AJ Schipani E. Role of carcinoma-associated fibroblasts and hypoxia in tumor progression. Curr Top Microbiol Immunol. 2010;345:31–45. 10.1007/82_2010_73 20517716

[178] Ziani L Buart S Chouaib S Thiery J. Hypoxia increases melanoma-associated fibroblasts immunosuppressive potential and inhibitory effect on T cell-mediated cytotoxicity. Oncoimmunology. 2021;10:1950953. 10.1080/2162402X.2021.1950953 34367731 PMC8312612

[179] Chen JY Li CF Kuo CC Tsai KK Hou MF Hung WC. Cancer/stroma interplay via cyclooxygenase-2 and indoleamine 2,3-dioxygenase promotes breast cancer progression. Breast Cancer Res. 2014;16:410. 10.1186/s13058-014-0410-1 25060643 PMC4220086

[180] Meisel R Zibert A Laryea M Göbel U Däubener W Dilloo D. Human bone marrow stromal cells inhibit allogeneic T-cell responses by indoleamine 2,3-dioxygenase-mediated tryptophan degradation. Blood. 2004;103:4619–21. 10.1182/blood-2003-11-3909 15001472

[181] Fallarino F Grohmann U Vacca C Bianchi R Orabona C Spreca A T cell apoptosis by tryptophan catabolism. Cell Death Differ. 2002;9:1069–77. 10.1038/sj.cdd.4401073 12232795

[182] Platten M Wick W Van den Eynde BJ. Tryptophan catabolism in cancer: beyond IDO and tryptophan depletion. Cancer Res. 2012;72:5435–40. 10.1158/0008-5472.CAN-12-0569 23090118

[183] Timosenko E Hadjinicolaou AV Cerundolo V. Modulation of cancer-specific immune responses by amino acid degrading enzymes. Immunotherapy. 2017;9:83–97. 10.2217/imt-2016-0118 28000524

[184] Ino Y Yamazaki-Itoh R Oguro S Shimada K Kosuge T Zavada J Arginase II expressed in cancer-associated fibroblasts indicates tissue hypoxia and predicts poor outcome in patients with pancreatic cancer. PLoS One. 2013;8:e55146. 10.1371/journal.pone.0055146 23424623 PMC3570471

[185] Becker LM O’Connell JT Vo AP Cain MP Tampe D Bizarro L Epigenetic reprogramming of cancer-associated fibroblasts deregulates glucose metabolism and facilitates progression of breast cancer. Cell Rep. 2020;31:107701. 10.1016/j.celrep.2020.107701 32492417 PMC7339325

[186] Fiaschi T Marini A Giannoni E Taddei ML Gandellini P De Donatis A Reciprocal metabolic reprogramming through lactate shuttle coordinately influences tumor-stroma interplay. Cancer Res. 2012;72:5130–40. 10.1158/0008-5472.CAN-12-1949 22850421

[187] Comito G Iscaro A Bacci M Morandi A Ippolito L Parri M Lactate modulates CD4^+^ T-cell polarization and induces an immunosuppressive environment, which sustains prostate carcinoma progression via TLR8/miR21 axis. Oncogene. 2019;38:3681–95. 10.1038/s41388-019-0688-7 30664688

[188] de Lourdes Mora-García M García-Rocha R Morales-Ramírez O Montesinos JJ Weiss-Steider B Hernández-Montes J Mesenchymal stromal cells derived from cervical cancer produce high amounts of adenosine to suppress cytotoxic T lymphocyte functions. J Transl Med. 2016;14:302. 10.1186/s12967-016-1057-8 27782859 PMC5080842

[189] Yu M Guo G Huang L Deng L Chang CS Achyut BR CD73 on cancer-associated fibroblasts enhanced by the A2B-mediated feedforward circuit enforces an immune checkpoint. Nat Commun. 2020;11:515. 10.1038/s41467-019-14060-x 31980601 PMC6981126

[190] Magagna I Gourdin N Kieffer Y Licaj M Mhaidly R Andre P CD73-mediated immunosuppression is linked to a specific fibroblast population that paves the way for new therapy in breast cancer. Cancers (Basel). 2021;13:5878. 10.3390/cancers13235878 34884993 PMC8657241

[191] O’Connor RA Chauhan V Mathieson L Titmarsh H Koppensteiner L Young I T cells drive negative feedback mechanisms in cancer associated fibroblasts, promoting expression of co-inhibitory ligands, CD73 and IL-27 in non-small cell lung cancer. Oncoimmunology. 2021;10:1940675. 10.1080/2162402X.2021.1940675 34290905 PMC8274440

[192] Zhu Y Shi C Zeng L Liu G Jiang W Zhang X High COX-2 expression in cancer-associated fibiroblasts contributes to poor survival and promotes migration and invasiveness in nasopharyngeal carcinoma. Mol Carcinog. 2020;59:265–80. 10.1002/mc.23150 31867776 PMC7027878

[193] Elwakeel E Brüne B Weigert A. PGE_2_ in fibrosis and cancer: insights into fibroblast activation. Prostaglandins Other Lipid Mediat. 2019;143:106339. 10.1016/j.prostaglandins.2019.106339 31100473

[194] Wang D DuBois RN. The role of prostaglandin E_2_ in tumor-associated immunosuppression. Trends Mol Med. 2016;22:1–3. 10.1016/j.molmed.2015.11.003 26711015 PMC4762482

[195] Gorchs L Fernández Moro C Bankhead P Kern KP Sadeak I Meng Q Human pancreatic carcinoma-associated fibroblasts promote expression of co-inhibitory markers on CD4^+^ and CD8^+^ T-Cells. Front Immunol. 2019;10:847. 10.3389/fimmu.2019.00847 31068935 PMC6491453

[196] Cremasco V Astarita JL Grauel AL Keerthivasan S MacIsaac K Woodruff MC FAP delineates heterogeneous and functionally divergent stromal cells in immune-excluded breast tumors. Cancer Immunol Res. 2018;6:1472–85. 10.1158/2326-6066.CIR-18-0098 30266714 PMC6597261

[197] Lakins MA Ghorani E Munir H Martins CP Shields JD. Cancer-associated fibroblasts induce antigen-specific deletion of CD8^+^ T cells to protect tumour cells. Nat Commun. 2018;9:948. 10.1038/s41467-018-03347-0 29507342 PMC5838096

[198] He XJ Tao HQ Hu ZM Ma YY Xu J Wang HJ Expression of galectin-1 in carcinoma-associated fibroblasts promotes gastric cancer cell invasion through upregulation of integrin β1. Cancer Sci. 2014;105:1402–10. 10.1111/cas.12539 25230369 PMC4462364

[199] Tang D Gao J Wang S Ye N Chong Y Huang Y Cancer-associated fibroblasts promote angiogenesis in gastric cancer through galectin-1 expression. Tumour Biol. 2016;37:1889–99. 10.1007/s13277-015-3942-9 26323258

[200] Rabinovich GA Toscano MA. Turning ‘sweet’ on immunity: galectin-glycan interactions in immune tolerance and inflammation. Nat Rev Immunol. 2009;9:338–52. 10.1038/nri2536 19365409

[201] Perillo NL Pace KE Seilhamer JJ Baum LG. Apoptosis of T cells mediated by galectin-1. Nature. 1995;378:736–9. 10.1038/378736a0 7501023

[202] Stillman BN Hsu DK Pang M Brewer CF Johnson P Liu FT Galectin-3 and galectin-1 bind distinct cell surface glycoprotein receptors to induce T cell death. J Immunol. 2006;176:778–89. 10.4049/jimmunol.176.2.778 16393961

[203] Gabrilovich DI Nagaraj S. Myeloid-derived suppressor cells as regulators of the immune system. Nat Rev Immunol. 2009;9:162–74. 10.1038/nri2506 19197294 PMC2828349

[204] Kumar V Patel S Tcyganov E Gabrilovich DI. The nature of myeloid-derived suppressor cells in the tumor microenvironment. Trends Immunol. 2016;37:208–20. 10.1016/j.it.2016.01.004 26858199 PMC4775398

[205] Kim JH Oh SH Kim EJ Park SJ Hong SP Cheon JH The role of myofibroblasts in upregulation of S100A8 and S100A9 and the differentiation of myeloid cells in the colorectal cancer microenvironment. Biochem Biophys Res Commun. 2012;423:60–6. 10.1016/j.bbrc.2012.05.081 22634002

[206] Zhao Q Huang L Qin G Qiao Y Ren F Shen C Cancer-associated fibroblasts induce monocytic myeloid-derived suppressor cell generation via IL-6/exosomal miR-21-activated STAT3 signaling to promote cisplatin resistance in esophageal squamous cell carcinoma. Cancer Lett. 2021;518:35–48. 10.1016/j.canlet.2021.06.009 34139285

[207] Deng Y Cheng J Fu B Liu W Chen G Zhang Q Hepatic carcinoma-associated fibroblasts enhance immune suppression by facilitating the generation of myeloid-derived suppressor cells. Oncogene. 2017;36:1090–101. 10.1038/onc.2016.273 27593937

[208] Yang X Lin Y Shi Y Li B Liu W Yin W FAP promotes immunosuppression by cancer-associated fibroblasts in the tumor microenvironment via STAT3-CCL2 signaling. Cancer Res. 2016;76:4124–35. 10.1158/0008-5472.CAN-15-2973 27216177

[209] Xiang H Ramil CP Hai J Zhang C Wang H Watkins AA Cancer-associated fibroblasts promote immunosuppression by inducing ROS-generating monocytic MDSCs in lung squamous cell carcinoma. Cancer Immunol Res. 2020;8:436–50. 10.1158/2326-6066.CIR-19-0507 32075803

[210] Allaoui R Bergenfelz C Mohlin S Hagerling C Salari K Werb Z Cancer-associated fibroblast-secreted CXCL16 attracts monocytes to promote stroma activation in triple-negative breast cancers. Nat Commun. 2016;7:13050. 10.1038/ncomms13050 27725631 PMC5062608

[211] Sharpe AH Pauken KE. The diverse functions of the PD1 inhibitory pathway. Nat Rev Immunol. 2018;18:153–67. 10.1038/nri.2017.108 28990585

[212] Bardhan K Anagnostou T Boussiotis VA. The PD1:PD-L1/2 pathway from discovery to clinical implementation. Front Immunol. 2016;7:550. 10.3389/fimmu.2016.00550 28018338 PMC5149523

[213] Khalili JS Liu S Rodríguez-Cruz TG Whittington M Wardell S Liu C Oncogenic BRAF(V600E) promotes stromal cell-mediated immunosuppression via induction of interleukin-1 in melanoma. Clin Cancer Res. 2012;18:5329–40. 10.1158/1078-0432.CCR-12-1632 22850568 PMC3463754

[214] Nazareth MR Broderick L Simpson-Abelson MR Kelleher RJ Jr Yokota SJ Bankert RB. Characterization of human lung tumor-associated fibroblasts and their ability to modulate the activation of tumor-associated T cells. J Immunol. 2007;178:5552–62. 10.4049/jimmunol.178.9.5552 17442937

[215] Pinchuk IV Saada JI Beswick EJ Boya G Qiu SM Mifflin RC PD-1 ligand expression by human colonic myofibroblasts/fibroblasts regulates CD4^+^ T-cell activity. Gastroenterology. 2008;135:1228–37.e2. 10.1053/j.gastro.2008.07.016 18760278 PMC2584612

[216] Zhang S Zhou C Zhang D Huang Z Zhang G. The anti-apoptotic effect on cancer-associated fibroblasts of B7-H3 molecule enhancing the cell invasion and metastasis in renal cancer. Onco Targets Ther. 2019;12:4119–27. 10.2147/OTT.S201121 31213832 PMC6538013

[217] Li Z Zhou J Zhang J Li S Wang H Du J. Cancer-associated fibroblasts promote PD-L1 expression in mice cancer cells via secreting CXCL5. Int J Cancer. 2019;145:1946–57. 10.1002/ijc.32278 30873585 PMC6767568

[218] Inoue C Miki Y Saito R Hata S Abe J Sato I PD-L1 induction by cancer-associated fibroblast-derived factors in lung adenocarcinoma cells. Cancers (Basel). 2019;11:1257. 10.3390/cancers11091257 31462002 PMC6770125

[219] Dou D Ren X Han M Xu X Ge X Gu Y Cancer-associated fibroblasts-derived exosomes suppress immune cell function in breast cancer via the miR-92/PD-L1 pathway. Front Immunol. 2020;11:2026. 10.3389/fimmu.2020.02026 33162971 PMC7581790

[220] Saw PE Chen J Song E. Targeting CAFs to overcome anticancer therapeutic resistance. Trends Cancer. 2022;8:527–55. 10.1016/j.trecan.2022.03.001 35331673

[221] De P Aske J Sulaiman R Dey N. Bête noire of chemotherapy and targeted therapy: CAF-mediated resistance. Cancers (Basel). 2022;14:1519. 10.3390/cancers14061519 35326670 PMC8946545

[222] Kraman M Bambrough PJ Arnold JN Roberts EW Magiera L Jones JO Suppression of antitumor immunity by stromal cells expressing fibroblast activation protein-alpha. Science. 2010;330:827–30. 10.1126/science.1195300 21051638

[223] Zhang Y Ertl HC. Depletion of FAP^+^ cells reduces immunosuppressive cells and improves metabolism and functions CD8^+^T cells within tumors. Oncotarget. 2016;7:23282–99. 10.18632/oncotarget.7818 26943036 PMC5029626

[224] Jiang GM Xu W Du J Zhang KS Zhang QG Wang XW The application of the fibroblast activation protein alpha-targeted immunotherapy strategy. Oncotarget. 2016;7:33472–82. 10.18632/oncotarget.8098 26985769 PMC5078111

[225] Busek P Mateu R Zubal M Kotackova L Sedo A. Targeting fibroblast activation protein in cancer - prospects and caveats. Front Biosci (Landmark Ed). 2018;23:1933–68. 10.2741/4682 29772538

[226] Loeffler M Krüger JA Niethammer AG Reisfeld RA. Targeting tumor-associated fibroblasts improves cancer chemotherapy by increasing intratumoral drug uptake. J Clin Invest. 2006;116:1955–62. Erratum in: J Clin Invest. 2009;119:421. 10.1172/JCI26532 16794736 PMC1481657

[227] Chen M Xiang R Wen Y Xu G Wang C Luo S A whole-cell tumor vaccine modified to express fibroblast activation protein induces antitumor immunity against both tumor cells and cancer-associated fibroblasts. Sci Rep. 2015;5:14421. Erratum in: Sci Rep. 2017;7:46841. Erratum in: Sci Rep. 2020;10:15609. 10.1038/srep14421 26394925 PMC4585784

[228] Duperret EK Trautz A Ammons D Perales-Puchalt A Wise MC Yan J Alteration of the tumor stroma using a consensus DNA vaccine targeting fibroblast activation protein (FAP) synergizes with antitumor vaccine therapy in mice. Clin Cancer Res. 2018;24:1190–201. 10.1158/1078-0432.CCR-17-2033 29269377 PMC5844837

[229] Xia Q Zhang FF Geng F Liu CL Wang YQ Xu P Improvement of anti-tumor immunity of fibroblast activation protein alpha based vaccines by combination with cyclophosphamide in a murine model of breast cancer. Cell Immunol. 2016;310:89–98. 10.1016/j.cellimm.2016.08.006 27545090

[230] Gottschalk S Yu F Ji M Kakarla S Song XT. A vaccine that co-targets tumor cells and cancer associated fibroblasts results in enhanced antitumor activity by inducing antigen spreading. PLoS One. 2013;8:e82658. 10.1371/journal.pone.0082658 24349329 PMC3861387

[231] Qian L Tang Z Yin S Mo F Yang X Hou X Fusion of dendritic cells and cancer-associated fibroblasts for activation of anti-tumor cytotoxic T lymphocytes. J Biomed Nanotechnol. 2018;14:1826–35. 10.1166/jbn.2018.2616 30041728

[232] Hu S Ma J Su C Chen Y Shu Y Qi Z Engineered exosome-like nanovesicles suppress tumor growth by reprogramming tumor microenvironment and promoting tumor ferroptosis. Acta Biomater. 2021;135:567–81. 10.1016/j.actbio.2021.09.003 34506976

[233] Kakarla S Chow KK Mata M Shaffer DR Song XT Wu MF Antitumor effects of chimeric receptor engineered human T cells directed to tumor stroma. Mol Ther. 2013;21:1611–20. 10.1038/mt.2013.110 23732988 PMC3734659

[234] Lo A Wang LS Scholler J Monslow J Avery D Newick K Tumor-promoting desmoplasia is disrupted by depleting FAP-expressing stromal cells. Cancer Res. 2015;75:2800–10. 10.1158/0008-5472.CAN-14-3041 25979873 PMC4506263

[235] Wang LC Lo A Scholler J Sun J Majumdar RS Kapoor V Targeting fibroblast activation protein in tumor stroma with chimeric antigen receptor T cells can inhibit tumor growth and augment host immunity without severe toxicity. Cancer Immunol Res. 2014;2:154–66. 10.1158/2326-6066.CIR-13-0027 24778279 PMC4007316

[236] Schuberth PC Hagedorn C Jensen SM Gulati P van den Broek M Mischo A Treatment of malignant pleural mesothelioma by fibroblast activation protein-specific re-directed T cells. J Transl Med. 2013;11:187. 10.1186/1479-5876-11-187 23937772 PMC3751305

[237] Bughda R Dimou P D’Souza RR Klampatsa A. Fibroblast activation protein (FAP)-targeted CAR-T cells: launching an attack on tumor stroma. Immunotargets Ther. 2021;10:313–23. 10.2147/ITT.S291767 34386436 PMC8354246

[238] Waldhauer I Gonzalez-Nicolini V Freimoser-Grundschober A Nayak TK Fahrni L Hosse RJ Simlukafusp alfa (FAP-IL2v) immunocytokine is a versatile combination partner for cancer immunotherapy. MAbs. 2021;13:1913791. 10.1080/19420862.2021.1913791 33974508 PMC8115765

[239] Brünker P Wartha K Friess T Grau-Richards S Waldhauer I Koller CF RG7386, a novel tetravalent FAP-DR5 antibody, effectively triggers FAP-dependent, avidity-driven DR5 hyperclustering and tumor cell apoptosis. Mol Cancer Ther. 2016;15:946–57. 10.1158/1535-7163.MCT-15-0647 27037412

[240] Trüb M Uhlenbrock F Claus C Herzig P Thelen M Karanikas V Fibroblast activation protein-targeted-4-1BB ligand agonist amplifies effector functions of intratumoral T cells in human cancer. J Immunother Cancer. 2020;8:e000238. 10.1136/jitc-2019-000238 32616554 PMC7333869

[241] Claus C Ferrara C Xu W Sam J Lang S Uhlenbrock F Tumor-targeted 4-1BB agonists for combination with T cell bispecific antibodies as off-the-shelf therapy. Sci Transl Med. 2019;11:eaav5989. 10.1126/scitranslmed.aav5989 31189721 PMC7181714

[242] Narra K Mullins SR Lee HO Strzemkowski-Brun B Magalong K Christiansen VJ Phase II trial of single agent Val-boroPro (talabostat) inhibiting fibroblast activation protein in patients with metastatic colorectal cancer. Cancer Biol Ther. 2007;6:1691–9. 10.4161/cbt.6.11.4874 18032930

[243] Eager RM Cunningham CC Senzer NN Stephenson J Jr Anthony SP O’Day SJ Phase II assessment of talabostat and cisplatin in second-line stage IV melanoma. BMC Cancer. 2009;9:263. 10.1186/1471-2407-9-263 19643020 PMC2731782

[244] Fang J Xiao L Joo KI Liu Y Zhang C Liu S A potent immunotoxin targeting fibroblast activation protein for treatment of breast cancer in mice. Int J Cancer. 2016;138:1013–23. 10.1002/ijc.29831 26334777 PMC4715643

[245] Ostermann E Garin-Chesa P Heider KH Kalat M Lamche H Puri C Effective immunoconjugate therapy in cancer models targeting a serine protease of tumor fibroblasts. Clin Cancer Res. 2008;14:4584–92. 10.1158/1078-0432.CCR-07-5211 18628473

[246] de Sostoa J Fajardo CA Moreno R Ramos MD Farrera-Sal M Alemany R. Targeting the tumor stroma with an oncolytic adenovirus secreting a fibroblast activation protein-targeted bispecific T-cell engager. J Immunother Cancer. 2019;7:19. 10.1186/s40425-019-0505-4 30683154 PMC6347837

[247] Tansi FL Rüger R Böhm C Steiniger F Kontermann RE Teichgraeber UK Activatable bispecific liposomes bearing fibroblast activation protein directed single chain fragment/trastuzumab deliver encapsulated cargo into the nuclei of tumor cells and the tumor microenvironment simultaneously. Acta Biomater. 2017;54:281–93. 10.1016/j.actbio.2017.03.033 28347861

[248] Millul J Bassi G Mock J Elsayed A Pellegrino C Zana A An ultra-high-affinity small organic ligand of fibroblast activation protein for tumor-targeting applications. Proc Natl Acad Sci U S A. 2021;118:e2101852118. 10.1073/pnas.2101852118 33850024 PMC8072232

[249] Brennen WN Rosen DM Wang H Isaacs JT Denmeade SR. Targeting carcinoma-associated fibroblasts within the tumor stroma with a fibroblast activation protein-activated prodrug. J Natl Cancer Inst. 2012;104:1320–34. 10.1093/jnci/djs336 22911669 PMC3529592

[250] Li L Zhou S Lv N Zhen Z Liu T Gao S Photosensitizer-encapsulated ferritins mediate photodynamic therapy against cancer-associated fibroblasts and improve tumor accumulation of nanoparticles. Mol Pharm. 2018;15:3595–9. 10.1021/acs.molpharmaceut.8b00419 29966416 PMC6435375

[251] Kaps L Schuppan D. Targeting cancer associated fibroblasts in liver fibrosis and liver cancer using nanocarriers. Cells. 2020;9:2027. 10.3390/cells9092027 32899119 PMC7563527

[252] Nicolás-Boluda A Vaquero J Laurent G Renault G Bazzi R Donnadieu E Photothermal depletion of cancer-associated fibroblasts normalizes tumor stiffness in desmoplastic cholangiocarcinoma. ACS Nano. 2020;14:5738–53. 10.1021/acsnano.0c00417 32338871

[253] Katsube R Noma K Ohara T Nishiwaki N Kobayashi T Komoto S Fibroblast activation protein targeted near infrared photoimmunotherapy (NIR PIT) overcomes therapeutic resistance in human esophageal cancer. Sci Rep. 2021;11:1693. 10.1038/s41598-021-81465-4 33462372 PMC7814141

[254] Lagares D Santos A Grasberger PE Liu F Probst CK Rahimi RA Targeted apoptosis of myofibroblasts with the BH3 mimetic ABT-263 reverses established fibrosis. Sci Transl Med. 2017;9:eaal3765. 10.1126/scitranslmed.aal3765 29237758 PMC8520471

[255] Scott AM Wiseman G Welt S Adjei A Lee FT Hopkins W A phase I dose-escalation study of sibrotuzumab in patients with advanced or metastatic fibroblast activation protein-positive cancer. Clin Cancer Res. 2003;9:1639–47. 12738716

[256] Haubeiss S Schmid JO Mürdter TE Sonnenberg M Friedel G van der Kuip H Dasatinib reverses cancer-associated fibroblasts (CAFs) from primary lung carcinomas to a phenotype comparable to that of normal fibroblasts. Mol Cancer. 2010;9:168. 10.1186/1476-4598-9-168 20579391 PMC2907332

[257] Tran E Chinnasamy D Yu Z Morgan RA Lee CC Restifo NP Immune targeting of fibroblast activation protein triggers recognition of multipotent bone marrow stromal cells and cachexia. J Exp Med. 2013;210:1125–35. 10.1084/jem.20130110 23712432 PMC3674706

[258] Sherman MH Yu RT Engle DD Ding N Atkins AR Tiriac H Vitamin D receptor-mediated stromal reprogramming suppresses pancreatitis and enhances pancreatic cancer therapy. Cell. 2014;159:80–93. 10.1016/j.cell.2014.08.007 25259922 PMC4177038

[259] Gorchs L Ahmed S Mayer C Knauf A Fernández Moro C Svensson M The vitamin D analogue calcipotriol promotes an anti-tumorigenic phenotype of human pancreatic CAFs but reduces T cell mediated immunity. Sci Rep. 2020;10:17444. 10.1038/s41598-020-74368-3 33060625 PMC7562723

[260] Santos AM Jung J Aziz N Kissil JL Puré E. Targeting fibroblast activation protein inhibits tumor stromagenesis and growth in mice. J Clin Invest. 2009;119:3613–25. 10.1172/JCI38988 19920354 PMC2786791

[261] Catenacci DV Junttila MR Karrison T Bahary N Horiba MN Nattam SR Randomized phase Ib/II study of gemcitabine plus placebo or vismodegib, a hedgehog pathway inhibitor, in patients with metastatic pancreatic cancer. J Clin Oncol. 2015;33:4284–92. 10.1200/JCO.2015.62.8719 26527777 PMC4678179

[262] Asif PJ Longobardi C Hahne M Medema JP. The role of cancer-associated fibroblasts in cancer invasion and metastasis. Cancers (Basel). 2021;13:4720. 10.3390/cancers13184720 34572947 PMC8472587

[263] Hanley CJ Mellone M Ford K Thirdborough SM Mellows T Frampton SJ Targeting the myofibroblastic cancer-associated fibroblast phenotype through inhibition of NOX4. J Natl Cancer Inst. 2018;110:109–20. 10.1093/jnci/djx121 28922779 PMC5903651

[264] Nishina T Takahashi S Iwasawa R Noguchi H Aoki M Doi T. Safety, pharmacokinetic, and pharmacodynamics of erdafitinib, a pan-fibroblast growth factor receptor (FGFR) tyrosine kinase inhibitor, in patients with advanced or refractory solid tumors. Invest New Drugs. 2018;36:424–34. 10.1007/s10637-017-0514-4 28965185

[265] Yao Y Guo Q Cao Y Qiu Y Tan R Yu Z Artemisinin derivatives inactivate cancer-associated fibroblasts through suppressing TGF-beta signaling in breast cancer. J Exp Clin Cancer Res. 2018;37:282. Erratum in: J Exp Clin Cancer Res. 2019;38:451. 10.1186/s13046-018-0960-7 30477536 PMC6258160

[266] Ohshio Y Teramoto K Hanaoka J Tezuka N Itoh Y Asai T Cancer-associated fibroblast-targeted strategy enhances antitumor immune responses in dendritic cell-based vaccine. Cancer Sci. 2015;106:134–42. 10.1111/cas.12584 25483888 PMC4399032

[267] Teixeira AF Ten Dijke P Zhu HJ. On-target anti-TGF-beta therapies are not succeeding in clinical cancer treatments: what are remaining challenges? Front Cell Dev Biol. 2020;8:605. 10.3389/fcell.2020.00605 32733895 PMC7360684

[268] Ciardiello D Elez E Tabernero J Seoane J. Clinical development of therapies targeting TGFbeta: current knowledge and future perspectives. Ann Oncol. 2020;31:1336–49. 10.1016/j.annonc.2020.07.009 32710930

[269] Johnson DE O’Keefe RA Grandis JR. Targeting the IL-6/JAK/STAT3 signalling axis in cancer. Nat Rev Clin Oncol. 2018;15:234–48. 10.1038/nrclinonc.2018.8 29405201 PMC5858971

[270] Allard B Pommey S Smyth MJ Stagg J. Targeting CD73 enhances the antitumor activity of anti-PD-1 and anti-CTLA-4 mAbs. Clin Cancer Res. 2013;19:5626–35. 10.1158/1078-0432.CCR-13-0545 23983257

[271] Willingham SB Ho PY Hotson A Hill C Piccione EC Hsieh J A2AR antagonism with CPI-444 induces antitumor responses and augments efficacy to anti-PD-(L)1 and anti-CTLA-4 in preclinical models. Cancer Immunol Res. 2018;6:1136–49. 10.1158/2326-6066.CIR-18-0056 30131376

[272] Tu E McGlinchey K Wang J Martin P Ching SL Floc’h N Anti-PD-L1 and anti-CD73 combination therapy promotes T cell response to EGFR-mutated NSCLC. JCI Insight. 2022;7:e142843. 10.1172/jci.insight.142843 35132961 PMC8855814

[273] Waickman AT Alme A Senaldi L Zarek PE Horton M Powell JD. Enhancement of tumor immunotherapy by deletion of the A2A adenosine receptor. Cancer Immunol Immunother. 2012;61:917–26. 10.1007/s00262-011-1155-7 22116345 PMC3589752

[274] Beavis PA Henderson MA Giuffrida L Mills JK Sek K Cross RS Targeting the adenosine 2A receptor enhances chimeric antigen receptor T cell efficacy. J Clin Invest. 2017;127:929–41. 10.1172/JCI89455 28165340 PMC5330718

[275] McCarthy JB El-Ashry D Turley EA. Hyaluronan, cancer-associated fibroblasts and the tumor microenvironment in malignant progression. Front Cell Dev Biol. 2018;6:48. Erratum in: Front Cell Dev Biol. 2018;6:112. 10.3389/fcell.2018.00048 29868579 PMC5951929

[276] Provenzano PP Cuevas C Chang AE Goel VK Von Hoff DD Hingorani SR. Enzymatic targeting of the stroma ablates physical barriers to treatment of pancreatic ductal adenocarcinoma. Cancer Cell. 2012;21:418–29. 10.1016/j.ccr.2012.01.007 22439937 PMC3371414

[277] Jacobetz MA Chan DS Neesse A Bapiro TE Cook N Frese KK Hyaluronan impairs vascular function and drug delivery in a mouse model of pancreatic cancer. Gut. 2013;62:112–20. 10.1136/gutjnl-2012-302529 22466618 PMC3551211

[278] Seki T Saida Y Kishimoto S Lee J Otowa Y Yamamoto K PEGPH20, a PEGylated human hyaluronidase, induces radiosensitization by reoxygenation in pancreatic cancer xenografts. A molecular imaging study. Neoplasia. 2022;30:100793. 10.1016/j.neo.2022.100793 35523073 PMC9079680

[279] Hingorani SR Harris WP Beck JT Berdov BA Wagner SA Pshevlotsky EM Phase Ib study of PEGylated recombinant human hyaluronidase and gemcitabine in patients with advanced pancreatic cancer. Clin Cancer Res. 2016;22:2848–54. 10.1158/1078-0432.CCR-15-2010 26813359 PMC7787348

[280] Van Cutsem E Tempero MA Sigal D Oh DY Fazio N Macarulla Tet al.; HALO 109-301 Investigators. Randomized phase III trial of pegvorhyaluronidase alfa with nab-paclitaxel plus gemcitabine for patients with hyaluronan-high metastatic pancreatic adenocarcinoma. J Clin Oncol. 2020;38:3185–94. 10.1200/JCO.20.00590 32706635 PMC7499614

[281] Chauhan VP Martin JD Liu H Lacorre DA Jain SR Kozin SV Angiotensin inhibition enhances drug delivery and potentiates chemotherapy by decompressing tumour blood vessels. Nat Commun. 2013;4:2516. 10.1038/ncomms3516 24084631 PMC3806395

[282] Spenlé C Saupe F Midwood K Burckel H Noel G Orend G. Tenascin-C: exploitation and collateral damage in cancer management. Cell Adh Migr. 2015;9:141–53. 10.1080/19336918.2014.1000074 25569113 PMC4422814

[283] Ni WD Yang ZT Cui CA Cui Y Fang LY Xuan YH. Tenascin-C is a potential cancer-associated fibroblasts marker and predicts poor prognosis in prostate cancer. Biochem Biophys Res Commun. 2017;486:607–12. 10.1016/j.bbrc.2017.03.021 28341124

[284] Brack SS Silacci M Birchler M Neri D. Tumor-targeting properties of novel antibodies specific to the large isoform of tenascin-C. Clin Cancer Res. 2006;12:3200–8. 10.1158/1078-0432.CCR-05-2804 16707621

[285] Mårlind J Kaspar M Trachsel E Sommavilla R Hindle S Bacci C Antibody-mediated delivery of interleukin-2 to the stroma of breast cancer strongly enhances the potency of chemotherapy. Clin Cancer Res. 2008;14:6515–24. 10.1158/1078-0432.CCR-07-5041 18927291

[286] Li ZL Zhang HL Huang Y Huang JH Sun P Zhou NN Autophagy deficiency promotes triple-negative breast cancer resistance to T cell-mediated cytotoxicity by blocking tenascin-C degradation. Nat Commun. 2020;11:3806. 10.1038/s41467-020-17395-y 32732922 PMC7393512

[287] Gieniec KA Butler LM Worthley DL Woods SL. Cancer-associated fibroblasts-heroes or villains? Br J Cancer. 2019;121:293–302. 10.1038/s41416-019-0509-3 31289350 PMC6738083

